# High Intraspecific mtDNA Polymorphisms and Mitonuclear Discordance: A Case Study of the *Cadlina laevis* (Linnaeus, 1767) Species Complex

**DOI:** 10.3390/ani16182934

**Published:** 2026-09-18

**Authors:** Irina Ekimova, Dmitry Fedorov, Darya Grishina, Dimitry Schepetov

**Affiliations:** 1 Invertebrate Zoology Department, Lomonosov Moscow State University, 119234 Moscow, Russia; irenekimova@gmail.com (I.E.); dairiagrishina00@gmail.com (D.G.); 2 MSU-SakhalinTech Biodiversity Research Laboratory, Sakhalin State University, 693008 Yuzhno-Sakhalinsk, Russia; 3 Center for Molecular and Cell Biology, 121205 Moscow, Russia; fedorov.kuleshov@gmail.com; 4 The Steinhardt Museum of Natural History, Israel National Center for Biodiversity Studies, Tel Aviv-Yafo 6997801, Israel

**Keywords:** mitochondrial genomes, species delimitation, phylogenetic analysis, Doridina, ITS, ribosomal DNA, barcoding, trans-Arctic, North Pacific, biodiversity

## Abstract

The *Cadlina laevis* (Linnaeus, 1767) species complex in the White Sea exhibits a high mitochondrial DNA polymorphism, with two divergent haplogroups (HA and HB) showing ~2.5% COI divergence, consistent with minimal interspecific mtDNA differences within this group. This study integrates complete mitochondrial genomes of both haplogroups of the White Sea *Cadlina laevis*, nuclear markers (18S, ITS1, ITS2, 28S, H3), and species-delimitation analyses to test whether mitochondrial lineages correspond to distinct species. Comparative mitogenomics revealed conserved gene order but heterogeneous divergence, with protein-coding genes showing p-distances from 1.0% (*COX2*) to 5.1% (*ATP6*) between haplogroups. Phylogenetic reconstructions based on nuclear markers consistently collapsed HA and HB into single, undifferentiated clade, demonstrating mitonuclear discordance. Similar patterns emerged for other mitochondrial lineages: *C. laevis* and North Pacific *C. vavilovi* Korshunova & Martynov, 2024, as well as *C. koltzovi* Korshunova & Martynov, 2024 and *Cadlina* sp. 3 showed no nuclear differentiation despite substantial COI p-distances. Species-delimitation methods yielded conflicting results, failing to provide consistent support for mitochondrial-defined species. The COI-wide distance distribution placed the HA–HB comparison within the “grey zone” of ambiguous species boundaries. The study demonstrates that mitochondrial data alone are unreliable for species delimitation in this group, suggesting that the current taxonomy may over-split mitochondrial lineages and require genome-wide nuclear evidence for robust taxonomic revision.

## Simple Summary

Sea slugs of the *Cadlina laevis* (Linnaeus, 1767) species complex from the White Sea display high variation in their mitochondrial DNA, making their taxonomy controversial. We analyzed complete mitochondrial genomes alongside multiple nuclear DNA markers, and applied contemporary species-delimitation methods. Our results revealed a clear disagreement between genetic datasets: while mitochondrial genomes differed noticeably, nuclear markers showed no such differentiation. Similar patterns were observed across other related *Cadlina* populations. These findings demonstrate that relying solely on mitochondrial DNA (such as standard DNA barcoding) is unreliable for identifying species in this group and can lead to over-splitting single species into many. Comprehensive nuclear genetic data is essential to achieve accurate taxonomic classification and avoid overestimating biodiversity.

## 1. Introduction

Contemporary integrative approach greatly streamlines taxonomic revisions. However, a growing number of described taxa and revisions makes erratic splitting or lumping a snowballing issue of further systematic updates. Decisions for groups with diverse morphology, partially sampled large geographic range, and a complex evolutionary history can be extremely challenging and should not rely on one-fits-all approaches [1]. Computational methods of species delimitation are an invaluable tool for guiding taxonomic revisions. In most cases such analysis helps to make a taxonomic decision that proves to hold well in further rounds of revision. However, these models rely on certain assumptions for the speciation scenario especially in approaches based on genetic distances, and may be susceptible to data sampling effects that are impossible to control in real life cases [2]. Thus, for peculiar cases where speciation occurred by not the most common path, their results may be contradictory or outright misleading. For example, in young species genetic distances are too small in comparison to other species of the genus or even absent [3,4]. Another set of issues arises from cases where due to certain scenarios (i.e., mitochondrial introgression or incomplete linage sorting) the use of only mitochondrial DNA-derived data gives extremely misleading results [5,6,7]. Even though they are rare such cases warrant explicit checks for mitonuclear discordance for all cases aiming at taxonomic reevaluations [8].

The *Cadlina laevis* species complex has been recently extensively revised in a series of papers [9,10,11]. In summary, this complex represents a high number of mitochondrial haplogroups differing in ~2.5–11% in Folmer’s fragment of *cytochrome c oxidase subunit I* (COI). At the same time, no strict and consistent morphological differences were found between most of these haplogroups [10,11]. As a result, two different interpretations have been suggested: (1) *C. laevis* represents a species complex and each haplogroup must be identified as a distinct species differing in fine-scale morphological features, or these features may partly overlap [9,10]; or (2) *C. laevis* represents a species complex, but some of these divergent haplogroups may represent an intraspecific diversity [11]. Most of the conclusions from the referred works were based mainly on mitochondrial DNA, while among nuclear markers only a short and highly conservative fragment of 28S rRNA was included in the phylogenetic framework [10]. Therefore, due to partly overlapping morphological features and the lack of information from nuclear markers, we presume that the hypothesis of 10 species differing in mtDNA only should be further tested using an integrative approach.

In this regard, the genetic diversity of *C. laevis* s.str. in the White Sea is of special interest. It was discovered recently that two divergent haplogroups are found in these waters [11] and uncorrected p-distance between them (from 2.29 to 2.54% in COI) are comparable with the minimal p-distances across NW Pacific clades interpreted as distinct species (e.g., between *C. franklinae* Korshunova & Martynov, 2024 and *C. paninae* Korshunova, Fletcher, Picton, Lundin, Kashio, N. Sanamyan, K. Sanamyan, Padula, Schrödl & Martynov, 2020, both from the Kurile Islands, see [10]). As the White Sea has a young geological age (it was formed due to marine transgression in warm conditions of the early Holocene, see [12]), the co-occurrence of several divergent haplogroups, one of which is endemic to this region, is not usual [13,14,15]. Therefore, a more in-depth study of the *Cadlina laevis* genetic diversity in the White Sea represents an important step forward for further revision of the whole complex. The objectives of this study are: (1) analysis of full mtDNA for the two White Sea haplogroups; (2) phylogenetic analyses of separate mitochondrial and nuclear genetic markers to test their congruence; (3) species-delimitation analyses based on different approaches; and (4) to estimate the divergence times within the *Cadlina laevis* species complex.

## 2. Materials and Methods

### 2.1. Material Collection

Specimens for this study (n = 53) were collected during various expeditions and field trips in the 2019–2022 period. Specimens from the White Sea (n = 16) were collected near the White Sea Biological Station (66°33′29.2″ N 33°06′19.6″ E) by scuba diving from 10 to 15 m depth. The nDNA data were additionally obtained for 37 specimens from the North-West Pacific (Table S1). Among them, 27 specimens were collected during 56th, 64th and 73rd cruises of R/V “Akademik Oparin” (Russia) to the Sea of Okhotsk and the Sea of Japan in 2019–2024 using an Agassiz trawl or by scuba diving. Five specimens were collected by scuba diving from Aniva Bay (Sakhalin Is.) and 5 specimens from Rudnaya Bay (the Sea of Japan). All specimens were preserved in 96% EtOH and stored at −20 °C to prevent DNA degradation. Voucher specimens are deposited to the collections of the National Scientific Center of Marine Biology (MIMB); DNA samples are stored at the Invertebrate Zoology Department, Lomonosov Moscow State University (Table S1).

### 2.2. DNA Extraction, PCR and Sequencing

Total genomic DNA was extracted from tissue samples preserved in 96% EtOH (Table S1) following the invertebrate protocol of the Canadian Center for DNA Barcoding [16]. For most specimens the partial mitochondrial *cytochrome c oxidase subunit I* (COI) was already sequenced and published [11,17], but three specimens (kur4–6, the Sea of Okhotsk) were barcoded for the first time. For all specimens we also performed the amplification of nuclear markers: partial histone *H3* (H3), 28S rRNA (28S), 18S rRNA (18S) and ribosomal internal transcribed spacer regions (ITS1, ITS2). For primer sequences and source see Supplementary Materials (Table S2). The amplification programs used are given in Table S2. Sequencing was performed with a NovaDye Terminator Cycle Sequencing Kit by GeneQuest (Moscow, Russia). Sequencing reactions were analyzed using an ABI 3500 Genetic Analyser (Applied Biosystems, Waltham, MA, USA) at the N.K. Koltsov Institute of Developmental Biology (Moscow, Russia) or Locus1616 at the Invertebrate Zoology Department, Lomonosov Moscow State University (Moscow, Russia). All novel sequences were submitted to NCBI GenBank (Table S1).

Two specimens representing divergent mtDNA haplogroups (wsbs1 and wsbs2) were used for mitochondrial genome sequencing. The extraction of DNA was performed using the same protocol as described above. Libraries construction was carried out according to the standard DNBSEQ short-read library preparation (BGI, Shenzhen, China) and further sequenced in paired-end mode with 150 bp read length on DNBSEQT7 platform (BGI, Shenzhen, China).

### 2.3. Mitochondrial Genome Assembly and Annotation

Paired-end Illumina libraries with a read length of 150 bp from each end (2 × 150 bp) were obtained for the two White Sea specimens, wsbs1 and wsbs2, representing two divergent mtDNA haplogroups. The quality of raw reads was assessed using FastQC v0.12.0. Paired reads were assembled de novo with SPAdes v3.15.5 [18] in paired-end genomic mode. The resulting circular mitochondrial assemblies were annotated using MITOS2 [19] under the invertebrate mitochondrial genetic code (NCBI translation). Assembly accuracy and local support were evaluated by mapping the original read pairs back to each assembled mitogenome with Bowtie2 v2.4.4 [20]. The resulting SAM/BAM files were sorted and indexed, and per-base depth and coverage profiles were inspected using SAMtools v1.3.5 [21].

### 2.4. Mitochondrial Genome Visualization and Comparative Analysis

Circular maps of the wsbs1 and wsbs2 mitogenomes were constructed in R with the *circlize* 0.4.18 package [22] using a custom script (Data S1). Genes were shown as directional arrows and coloured by respiratory-chain/RNA class. GC content was calculated in circular 200 bp windows advanced in 100 bp steps. AT-skew and GC-skew were calculated as (A − T)/(A + T) and (G − C)/(G + C), respectively.

Comparative mitogenome analysis included *Cadlina japonica* Baba, 1937 (NC_063124.1), *C. koreana* Do, Jung, Kil & Kim, 2020 (NC_063125.1), *C. umiushi* Korshunova, Picton, Sanamyan & Martynov, 2015 (NC_063126.1) [23], and the newly assembled *C. laevis* mitogenomes wsbs1 and wsbs2. Public FASTA sequences and annotations were downloaded from NCBI GenBank, whereas wsbs1 and wsbs2 annotations were obtained with MITOS2 [19]. All mitogenomes were oriented in the same direction and linearized at *COX1*, with the *Cadlina laevis* wsbs1 mitogenome used as the coordinate reference. Nucleotide p-distances between the reference and each of the four comparison genomes were calculated in 200 bp sliding windows advanced in 100 bp steps. For each window, the mean pairwise p-distance across the five mitogenomes was also calculated. Window-based distances were mapped onto the wsbs1 coordinates and visualized as a heatmap, while alignment gaps and local structural discontinuities were recorded separately. Gene coordinates and transcriptional orientations were obtained from the corresponding GFF annotations and plotted above the divergence profiles using the *gggenomes* v1.1.2 package [24]. The complete visualization was generated with a custom R script (Data S1).

### 2.5. Codon-Aware Alignment and Protein-Coding Gene p-Distances

Nucleotide sequences of all 13 mitochondrial protein-coding genes (*ATP6*, *ATP8*, *COX1*, *COX2*, *COX3*, *CYTB*, *ND1*, *ND2*, *ND3*, *ND4*, *ND4L*, *ND5* and *ND6*) were extracted from the five mitogenomes representing four species. Each gene was aligned separately at the codon level with MACSE v2 [25]. The invertebrate mitochondrial genetic code was selected with -gc_def 5.

Uncorrected pairwise nucleotide distances (p-distances, i.e., the proportion of different aligned positions) were calculated from each codon alignment with Goalign [26]. Gene-wise violin plots and overlaid points were produced in R with *tidyverse* v2.0.0 [27] and *ggplot2* 4.0.3 [28] using a custom script (Data S1). Between-species comparisons were plotted in black, whereas the wsbs1–wsbs2 comparison was highlighted in red.

### 2.6. Phylogenetic Analysis of Partial Markers

Raw reads for each partial marker from Sanger sequencing were assembled and checked for improper base calling in Geneious R10 (Biomatters, Auckland, New Zealand). Edited sequences were verified for contamination using the BLAST-n algorithm from BLAST+ 2.17.0, run over the NCBI GenBank nr/nt database. For the phylogenetic reconstruction we used sequences from previously published revisions of *Cadlina laevis* species complex [9,10,11,17,23,29] (Table S1). Representatives of the genus *Aldisa* Bergh, 1878 were used as the outgroup [9]. Original data and publicly available sequences were aligned with the MUSCLE [30] algorithm implemented in MEGA 7 [31]. Sequences from protein-coding genes were translated into amino acids to eliminate potential pseudogene reads by verifying coding sequences reading frame integrity. No pseudogenes were detected.

The resulting alignments were of 656 bp for COI, 332 bp for H3, 1817 bp for 18S, 350 bp for 28S, and 582 bp for ITS1 and 586 bp for ITS2. Phylogenetic analyses were conducted for each marker separately using Bayesian and maximum-likelihood methods. The best-fitting nucleotide evolution model was tested in MEGA 7 [31] based on the Bayesian information criterion (BIC) for each marker; the chosen models are identified in Table S2. Bayesian analysis was conducted in MrBayes 3.2.7a [32]. Maximum-likelihood analyses were conducted in raxmlGUI 2.0 [33] with automatically estimated pseudoreplicate number defined by the autoMRE algorithm [34] under the GTRCAT approximation. The resulting phylogenetic trees were visualized in FigTree 1.4.4 [35] and then annotated in Adobe Illustrator 2020 [36]. In several cases one of three 18S fragments and spacer region showed low amplification success, or indel polymorphisms in spacer regions were detected after sequencing, so these data were not used for the phylogenetic reconstruction. Due to the unavailability of several *Cadlina* species in our material, for strict comparison with nDNA phylogenies the COI-based phylogenetic analysis was repeated for the reduced dataset, which included only specimens with available nDNA data (n = 51). For the partitioned analysis all markers (COI and nuclear gene fragments) were concatenated using a simple Biopython script [37]. Two maximum-likelihood reconstructions were done for different concatenated datasets: one included mitochondrial and nuclear markers, and the second included only nDNA dataset (excluding samples for which only 28S rRNA was obtained). The phylogenetic reconstructions of both datasets were done in the HPC-PTHREADS-AVX version of RaxML [38] with GTRCAT approximation applied to all markers independently and rapid bootstrapping [39] with 600 pseudoreplicates for the dataset of all studied markers and 1000 for the nDNA dataset as determined by AutoMRE algorithm.

### 2.7. Species Delimitation

Three molecular species-delimitation methods were used to confirm the status of recovered clades as putative candidate species. The Assemble Species by Automatic Partitioning (ASAP) analysis [40] was conducted in iTaxoTools 0.1 [41] based on the COI dataset with four available models: Jukes–Cantor (JC69), Kimura (K2P), Simple distance (SD) and Tamura–Nei (TN). Also, two separate analyses of Poisson Tree Processes (PTP) method based on the Maximum likelihood (mPTP) and Bayesian inference (bPTP) were conducted [42]. These tests were run in iTaxoTools 0.1 [43] based on modified COI dataset (excluding identical sequences) with 500,000 generations and with other settings (thinning, burn-in and seed) set as default. Additionally, we ran the General Mixed Yule–Coalescent (GMYC) test proposed by [43] and implemented by [44]. COI-based tree was calculated using BEAST 2.7 [45] and then analyzed in the R environment package *splits* 1.0, following [44]. Uncorrected p-distances were calculated in MEGA 7 [31].

### 2.8. COI-Wide Distance Distribution and Heatmap

For the broader COI comparison, we reused the alignment previously prepared for the phylogenetic reconstruction and species-delimitation analyses described above. It comprised 190 sequences (185 *Cadlina* and five *Aldisa* outgroups), corresponding to 17,955 unique unordered sequence pairs. Pairwise p-distances were computed with Goalign [26]. The five *Aldisa* outgroups were then excluded from the genus-level visualization, leaving 185 *Cadlina* sequences and 17,020 unique pairwise comparisons. The distribution was drawn as a violin with adjust = 1.1 (Data S1). Five density clusters were delimited objectively from a Gaussian kernel-density estimate calculated with stats::density (default bandwidth bw = “nrd0”, adjust = 1.1), matching the smoothing used for the violin. Local maxima were identified from changes in the sign of the first difference in the evaluated density curve; the four intervening local minima (*p* = 0.0216504, 0.0615604, 0.1012303 and 0.1434209) defined the five intervals. Species-pair categories were ranked by their frequency within each interval, and the five most frequent categories were displayed beside the corresponding bracket. All species-pair categories and all underlying comparisons are reported in Table S3.

A symmetric COI p-distance heatmap was generated in R with *tidyverse* [27] and *ggplot2* [28] using a custom script (Data S1). For this heatmap only, the singleton *C. rumia* and the strongly divergent South African sequence “*Cadlina* sp. 2 EU982727” were excluded, leaving 183 sequences; this filter was not applied to the violin/KDE analysis. Species represented by at least two sequences were ordered by average-linkage hierarchical clustering (hclust, method = “average”) of a species-level matrix containing the mean p-distance for every species pair. Each singleton species was then placed immediately after the non-singleton species to which it had the minimum observed sequence-level p-distance; ties were resolved alphabetically. Sequences were sorted by identifier within species, and rows and columns used the same order.

### 2.9. Divergence Times Estimation

To estimate divergence times of the *Cadlina laevis* species complex, a chronogram was reconstructed based on the COI dataset. The genus *Aldisa* was used as an outgroup [9]. The analysis was performed in BEAST 2.7 [45]. Since no fossil-based calibrations are available for nudibranchs, we used the putative evolutionary rate calculated for the trans-Arctic Gastropoda taxa by [46]: 6.1% My^−1^. This rate was estimated by a calculation of the TN+G pairwise distances across those trans-Arctic taxa that have invaded the Atlantic via the Bering Strait during the Great Trans-Arctic Interchange and this action was supported by paleontological data (*Littorina squalida* versus *L. littorea*). The Strict Clock molecular clock model was implemented in BEAST 2.7 [45] under the Birth–Death tree prior; the clock rate of 0.0307 was based on gastropod substitution rate of 6.1% My^−1^ as described above. Analysis was conducted with 10 million generations and a sampling frequency of 1000, with a burn-in of 25% of the trees generated. Convergence was assessed using Tracer 1.7.2 [47].

### 2.10. Taxonomic Framework and Clade Naming Conventions

In our taxonomic decisions we strive to follow the phylogenetic species concept (sensu Hennig & Wiley [48,49], with species defined as lineage segments between speciation events. We implement this in compliance with the latest recommendations for systematics and taxonomy of the group [50] by searching for reciprocal monophyly across known loci complemented with stable diagnostic traits in morphology. To name clades recovered in our phylogenetic reconstructions we used species names from the most recent work on *Cadlina laevis* species complex [10]. However, some of clades identified in [11] were not studied by the referred authors; in these cases we used “*Cadlina* sp. 1”, “*Cadlina* sp. 3” and “*Cadlina* sp. 7” as in [11] for clarity.

## 3. Results

### 3.1. Mitochondrial Genome Assembly and Comparative Analyses

**Figure 1:**
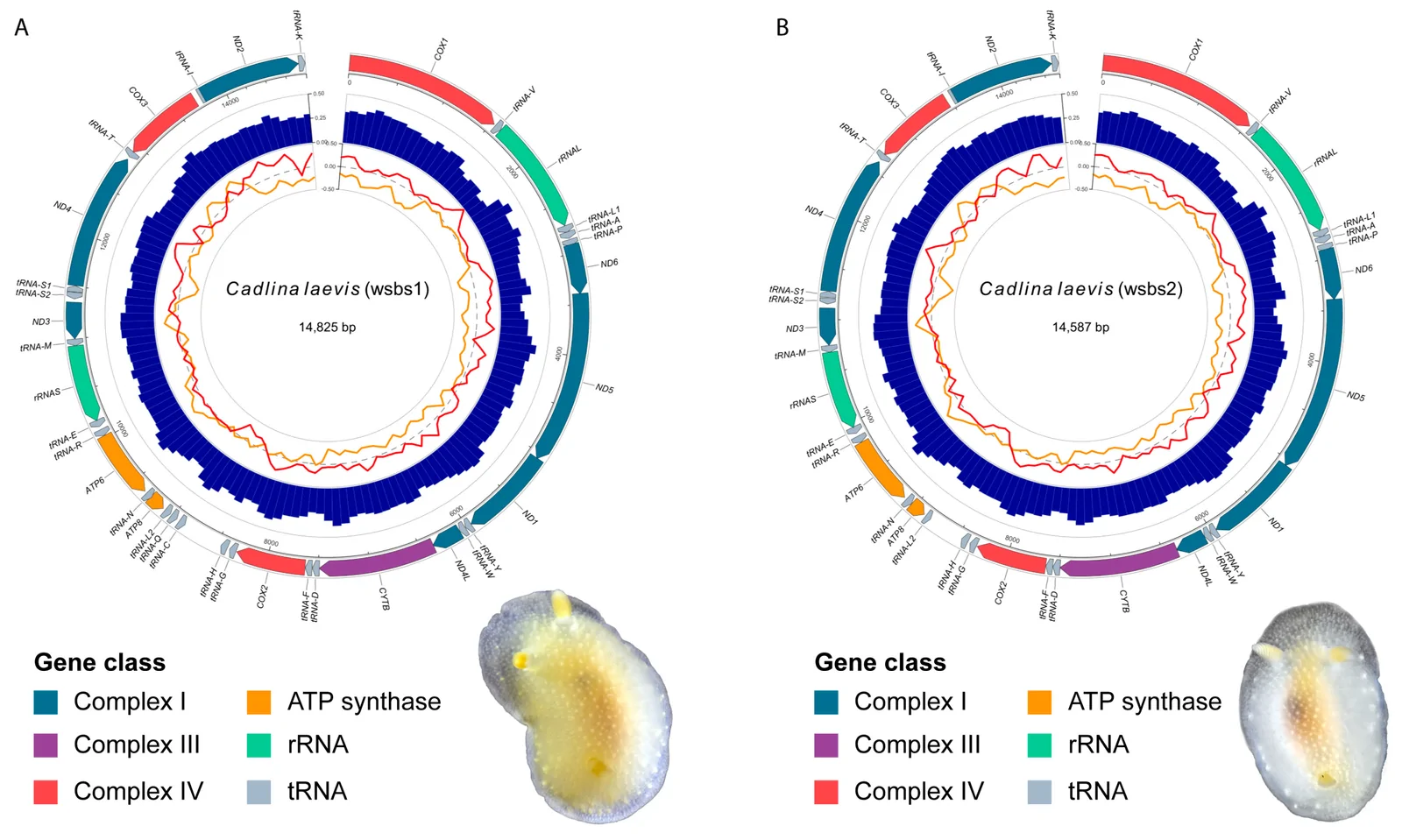
Circular mitochondrial genome maps of White Sea *Cadlina laevis* specimens: (**A**) wsbs1 (14,825 bp) and (**B**) wsbs2 (14,587 bp). The outer arrows indicate gene coordinates and transcriptional direction; colours identify respiratory-chain complexes, ATP synthase, rRNAs and tRNAs. The coordinate scale is shown in base pairs. Blue radial bars show GC content calculated in circular 200 bp windows at 100 bp steps. The red and orange inner lines show GC-skew and AT-skew, respectively, and the dashed grey circle marks zero skew.

The complete circular mitochondrial genomes assembled from the two White Sea specimens were 14,825 bp (wsbs1) and 14,587 bp (wsbs2), differing in total length by 238 bp. MITOS2 recovered the conventional 37-gene metazoan mitochondrial complement in both assemblies (13 protein-coding genes, 22 tRNAs and two rRNAs). The order and transcriptional orientation of the annotated genes were the same in the two specimens, whereas GC-content and nucleotide-skew profiles varied locally around the circle (Figure 1).

**Figure 2:**
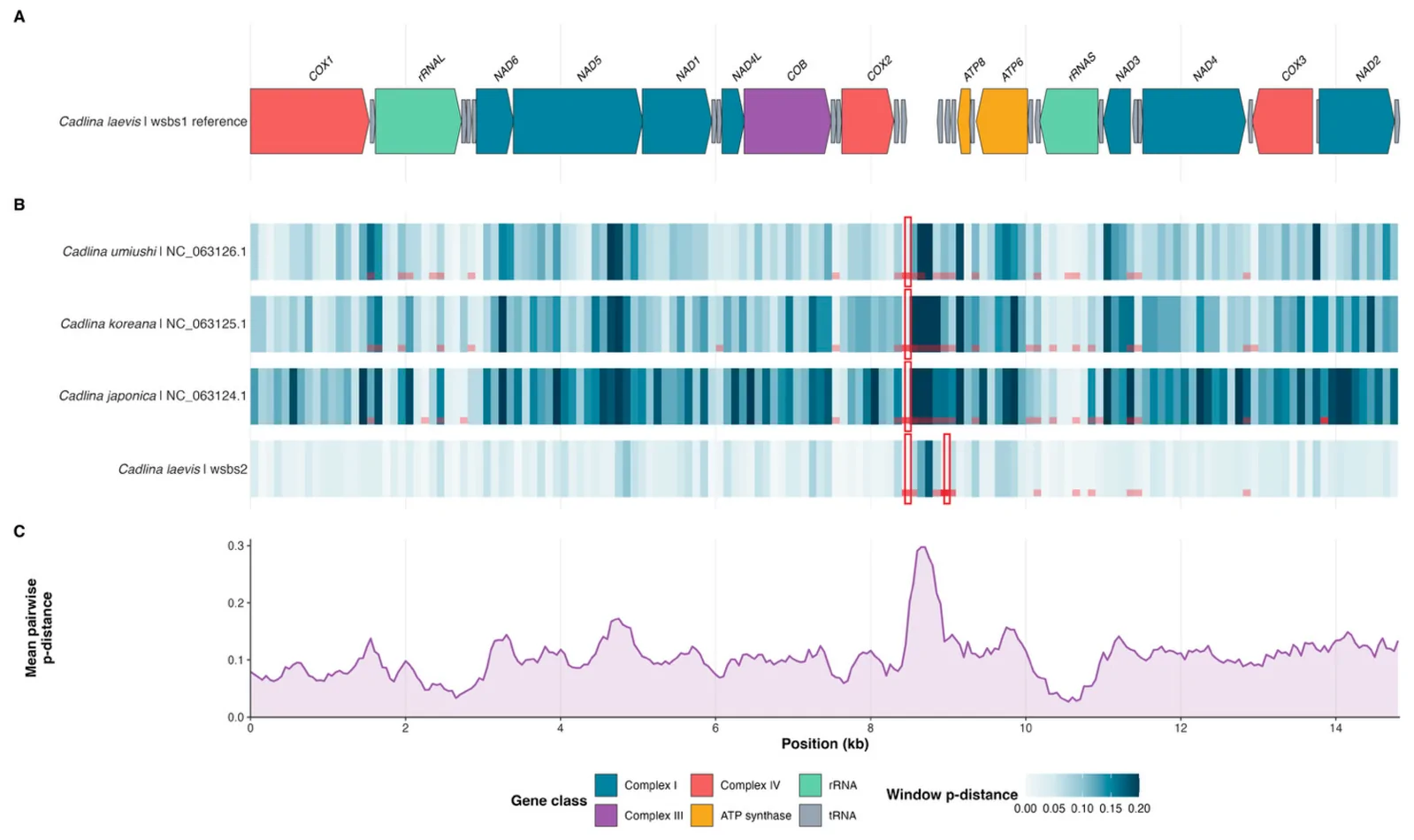
Comparative analysis of mitochondrial genome divergence in *Cadlina*. (**A**) Gene organization of the *Cadlina laevis* wsbs1 mitogenome used as the reference. Arrows show gene coordinates and transcriptional direction; colours denote gene classes. (**B**) Window-based nucleotide p-distances between the wsbs1 reference and the mitogenomes of *C. umiushi* (NC_063126.1), *C. koreana* (NC_063125.1), *C. japonica* (NC_063124.1), and *C. laevis* wsbs2. Increasing colour intensity indicates greater nucleotide divergence. Red outlines highlight the most prominent local discontinuities in the tRNA-rich region. (**C**) Mean window-based pairwise p-distance across the compared mitogenomes along the mitochondrial genome. All genomes were linearized at *COX1*; coordinates are shown in kilobases.

Comparison with the complete mitogenomes of *C. japonica*, *C. koreana* and *C. umiushi* demonstrated strongly conserved mitochondrial gene order. Reciprocal BLAST-n hits formed nearly continuous homologous blocks across the five genomes; the main local discontinuities occurred in short tRNA-rich intervals and near the ATP8–ATP6 and terminal COX3–ND2 regions. The largest mean p-distance of approximately 0.30 was detected at 8.5–9.0 kb. No large inversion or translocation was detected in either *C. laevis* mitogenome (Figure 2).

### 3.2. Protein-Coding Gene Divergence

**Figure 3:**
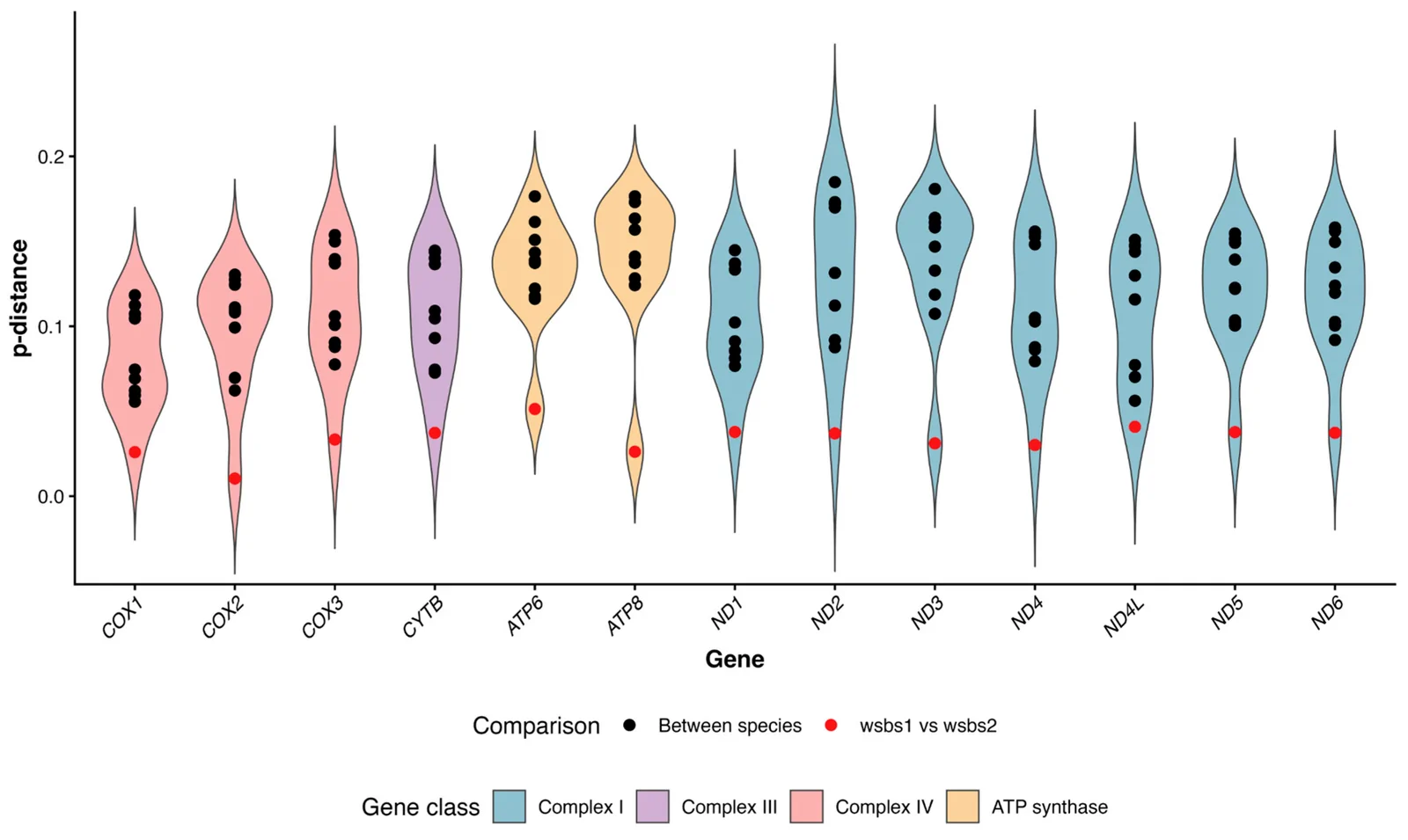
Uncorrected pairwise nucleotide distances for 13 mitochondrial protein-coding genes in five samples representing four *Cadlina* species. Each violin contains ten unique pairwise comparisons for one gene. Black points denote comparisons between species, and the red point denotes the wsbs1–wsbs2 comparison within *C. laevis*. Violin fill colours correspond to respiratory-chain complex or ATP-synthase gene classes.

The magnitude of nucleotide divergence differed markedly among the 13 protein-coding genes (Figure 3). In every gene, the wsbs1–wsbs2 comparison was smaller than all nine comparisons between species. Its p-distance (rounded to three decimals) ranged from 0.010 in *COX2* to 0.051 in *ATP6*; corresponding values were 0.026 for *COX1* and 0.033 for *COX3*. Between-species values reached 0.185 in *ND2* and 0.181 in *ND3*, illustrating pronounced gene-to-gene heterogeneity in mitochondrial sequence divergence.

### 3.3. Phylogenetic Reconstruction of COI Dataset

**Figure 4:**
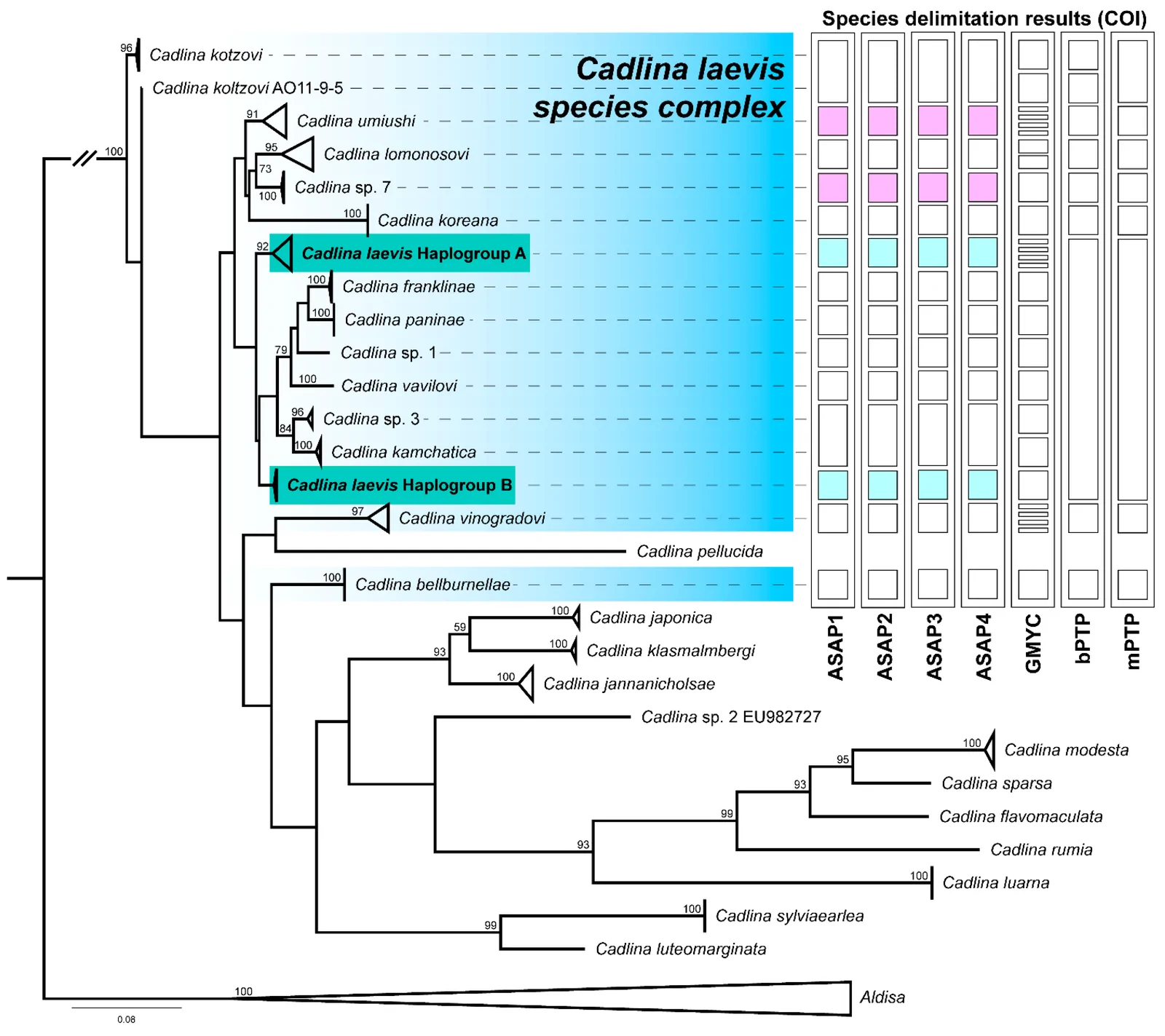
COI-based maximum-likelihood tree of the genus *Cadlina* and species-delimitation results. Green highlighting identifies two divergent White Sea lineages of *Cadlina laevis* s.str. Numbers in respective nodes indicate support from Maximum Likelihood (BS). Designations: ASAP1—Assemble Species by Automatic Partitioning (ASAP) with Jukes–Cantor model; ASAP2—same analysis, simple distance model; ASAP3—same analysis, Kimura-2P model; ASAP4—same analysis, Tamura–Nei model; GMYC—General Mixed Yule–Coalescent analysis; bPTP—Bayesian implementation of the Poisson Tree Processes; mPTP—Maximum-likelihood implementation of the Poisson Tree Processes. Blue-coloured (*Cadlina laevis* Haplogroups A and B) and pink-coloured blocks (*Cadlina umiushi* + *Cadlina* sp. 7) each indicate a single group in ASAP analyses. Only BS > 70 is shown.

The Maximum Likelihood (ML) and Bayesian (BI) analyses of COI alignment recovered different tree topologies, but the support values for most deep nodes were low in both methods (Figure 4 and Figure S1). The Maximum-Likelihood tree (Figure 4) identified 13 clades, corresponding to species *Cadlina koltzovi*, *C. umiushi*, *C. lomonosovi*, *Cadlina* sp. 7, *Cadlina koreana*, *C. laevis* Haplogroup A (HA), *C. franklinae*, *C. paninae*, *Cadlina* sp. 3, *C. kamchatica*, *C. laevis* Haplogroup B (HB), *C. vinogradovi*, *C. bellburnellae*. Most of these clades had the bootstrap support (BS) higher than 90, except *C. laevis* haplogroup B (BS = 69). Additionally, three derived singletons were identified: *Cadlina koltzovi* AO11-9-5, *Cadlina* sp. 1 and *C. vavilovi*. These 13 clades and three singletons represented the primary species hypothesis (PSH). The Maximum-likelihood analysis did not recover *Cadlina laevis* species complex as a monophyletic group, but this was not statistically supported.

The Bayesian tree of the COI dataset revealed the same clades as in ML analysis (Figure S1). The relationships between them were not resolved in most cases as in ML. In BI *C. laevis* HA and HB formed sister clades (not supported, PP = 0.71), as well as *C. koltzovi* ZMMU Op-840–855 and *C. koltzovi* AO11-9-5 (PP = 1). The *C. laevis* species complex was recovered monophyletic but not supported (PP = 0.71).

### 3.4. COI-Based Species Delimitation

As a primary species hypothesis (PSH) we used clades of *Cadlina laevis* species complex (n = 16) recovered in the phylogenetic analysis of mitochondrial marker COI (Figure 4). Results of COI-based species-delimitation analyses (ASAP, GMYC, bPTP and mPTP) gave conflicting species hypotheses compared to PSH and to each other (Data S2). The highest number of groups (n = 26) was recovered in the GMYC analysis. According to it, *C. umiushi*, *C. laevis* HA and *C. vinogradovi* split into four units each, and *C. lomonosovi* was represented by two units; other groups confirmed the PSH. Different models of ASAP analysis gave similar results with 12 operational units. Among them, eight clades from PSH were united into four groups: (1) *C. umiushi* and *Cadlina* sp. 7, (2) *Cadlina laevis* HA and HB, (3) *Cadlina koltzovi* ZMMU Op-840–855 and *C. koltzovi* sample AO11-9-5, and (4) *C. kamchatica* and *Cadlina* sp. 3. Finally, the smallest number of operational units were recovered in both PTP analyses: eight in mPTP and nine in bPTP. In this case, *C. franklinae*, *C. paninae*, *Cadlina* sp. 1, *C. vavilovi*, *Cadlina* sp. 3, *C. kamchatica* and both *C. laevis* haplogroups were united in a single molecular operational taxonomic unit (mOTU), and the differentiation between *Cadlina koltzovi* ZMMU Op-840–855 and *C. koltzovi* sample AO11-9-5 was recovered only in bPTP.

### 3.5. COI-Wide p-Distance Structure

**Figure 5:**
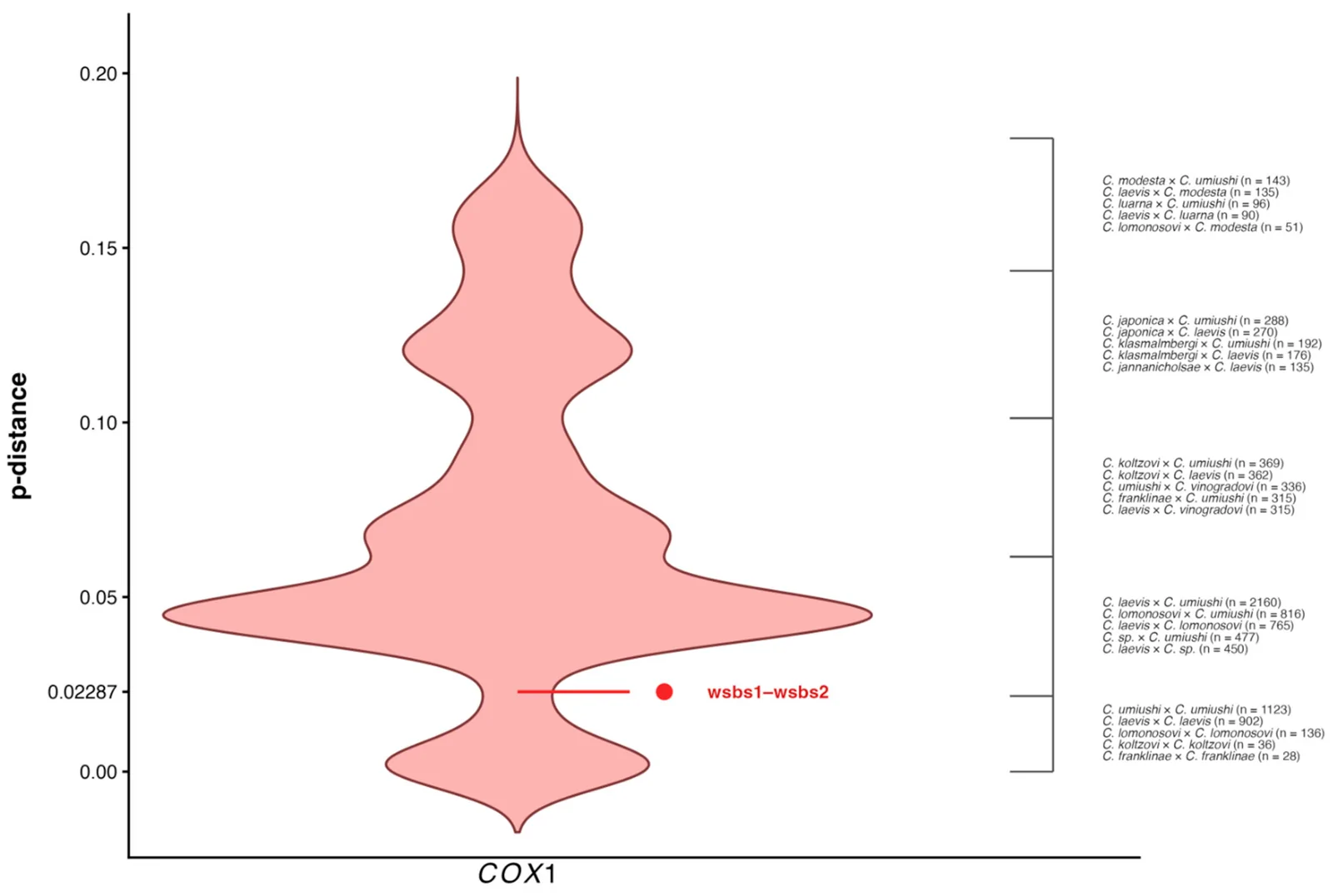
Distribution of 17,020 uncorrected COI (*COX1*) p-distances among 185 *Cadlina* sequences. The violin shows the Gaussian kernel-density estimate. The red point and horizontal marker identify the wsbs1–wsbs2 comparison (*p* = 0.0228659). Square brackets delimit five intervals using local minima of the same density curve (0.0216504, 0.0615604, 0.1012303 and 0.1434209). For readability, only the five most frequent species-pair categories in each interval are printed beside the graph; all categories and all individual comparisons are listed in Table S3.

The 17,020 COI comparisons among 185 *Cadlina* sequences formed five reproducible density modes centred at approximately 0.00209, 0.04488, 0.06738, 0.12062 and 0.15548 (Figure 5). The corresponding KDE-delimited intervals contained 2311, 6728, 3612, 2941 and 1428 comparisons, respectively. The lowest interval was dominated by within-species comparisons, whereas successively higher intervals contained progressively more distant combinations of named species (Table S3). The wsbs1–wsbs2 p-distance was 0.02287, immediately above the first density minimum (0.0216504) and therefore assigned to the second interval. It nevertheless remained within the observed *C. laevis* range (0–0.024390) and lay at approximately the 98th percentile of the 990 within-*C. laevis* comparisons.

**Figure 6:**
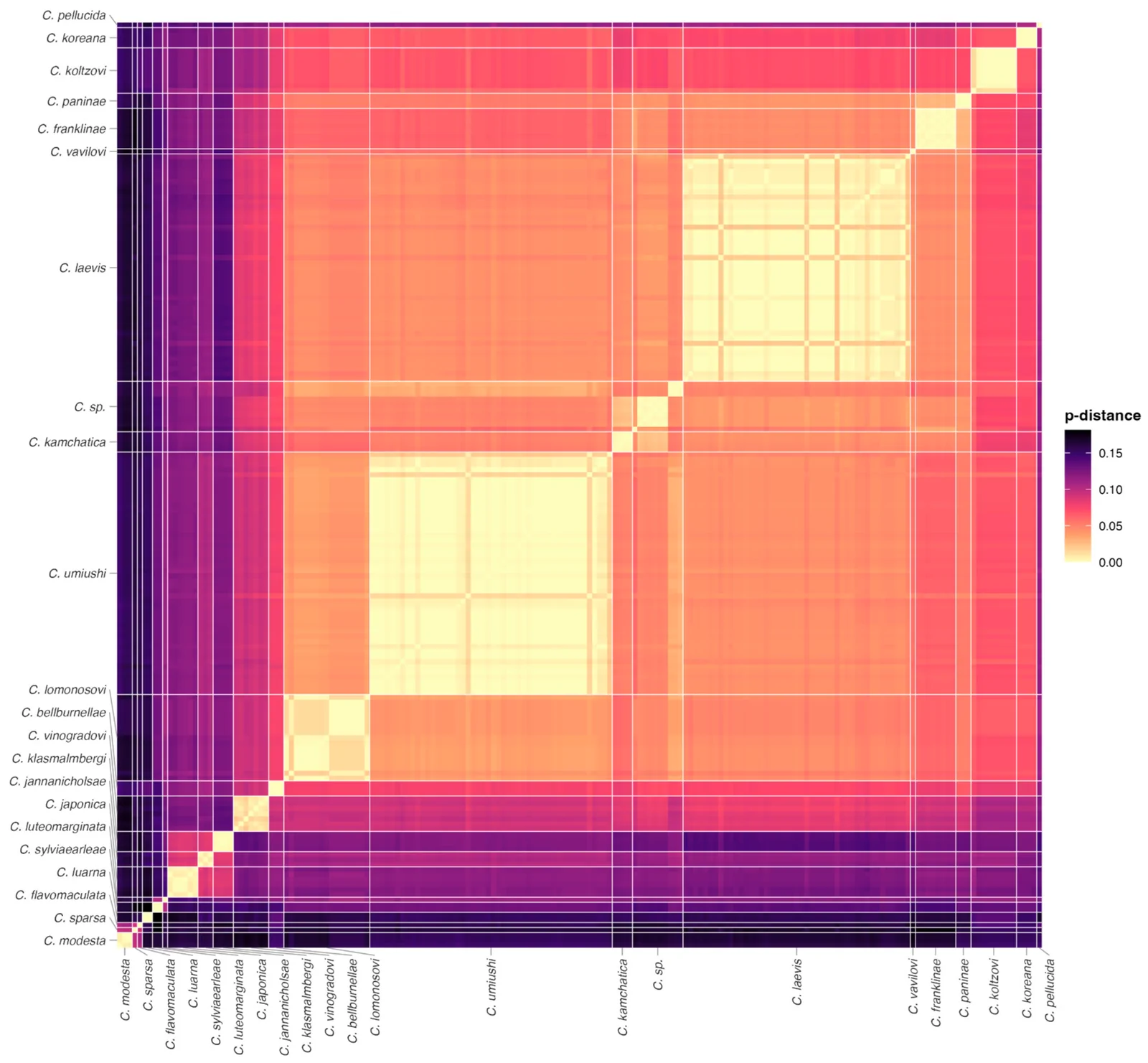
Symmetric heatmap of pairwise COI p-distances among 183 *Cadlina* sequences retained for heatmap visualization. Light colours indicate small and dark colours large p-distances; white lines delimit species blocks. Species with two or more sequences were ordered by average-linkage hierarchical clustering of their mean species-pair distance matrix. Each singleton species was placed immediately after the non-singleton species with the smallest observed sequence-level p-distance. Rows and columns use the same order. *Cadlina rumia* and *Cadlina* sp. 2 South_Africa_EU982727 were excluded from this heatmap only.

The ordered distance heatmap recovered compact low-distance diagonal blocks for the best-sampled species, together with several pale off-diagonal blocks that demonstrate overlap between some intra- and interspecific distance ranges (Figure 6). The large *C. laevis*, *C. umiushi* and *C. lomonosovi* blocks retained visible internal substructure, whereas comparisons involving the most divergent taxa formed dark rows and columns. Placing singleton species beside their nearest multi-sample species made these affinities visible without altering the underlying pairwise distances.

### 3.6. Phylogenetic Reconstructions of nDNA and Concatenated Datasets

**Figure 7:**
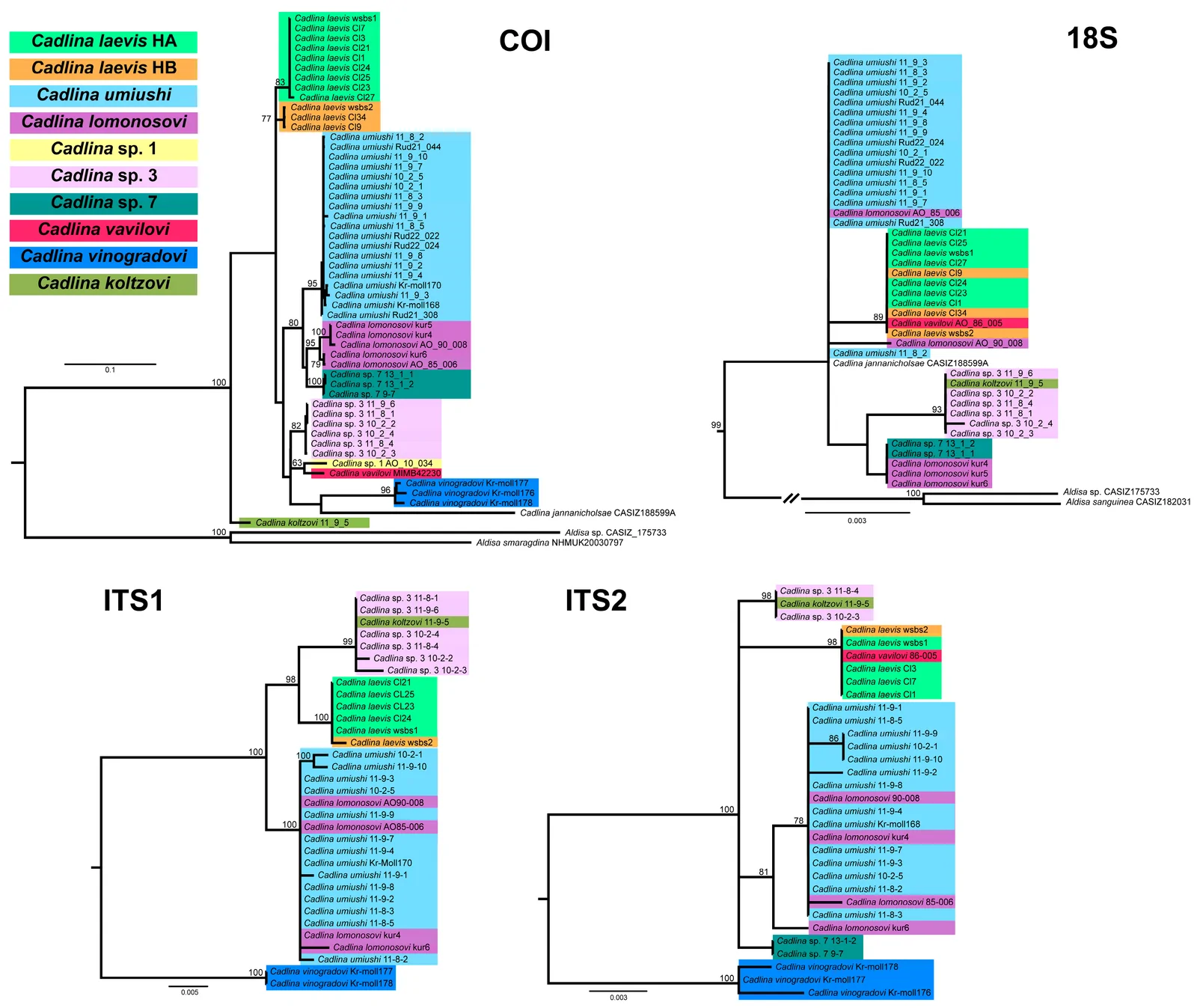
Maximum-likelihood trees of the genus *Cadlina laevis* complex based on mtDNA (COI) and nDNA (18S, ITS1, ITS2) data. Numbers in respective nodes indicate support from Maximum Likelihood (BS). Each group from initial species hypothesis is highlighted by specific colour. Representatives of the genus *Aldisa* are chosen as the outgroup. Only BS >70 is shown.

Phylogenetic trees based on 28S and H3 datasets showed no or little variation (Figure S2). The reconstructions based on 18S, ITS1 and ITS2 datasets had better resolution (Figure 7). The recovered topologies in these cases showed a clear mitonuclear discordance. On the 18S phylogenetic tree representatives of *C. umiushi*, *C. lomonosovi* (AO85-006) and *C. jannanicholsae* formed a basal polytomy, while *C. lomonosovi* (AO90-008) represented a derived singleton. *Cadlina laevis* HA and HB and *C. vavilovi* formed a single clade with no nucleotide variation (BS = 89). *Cadlina* sp. 3 and *C. koltzovi* were placed in a single group (BS = 93), similarly with *Cadlina* sp. 7 and *Cadlina lomonosovi* (kur4-kur6). The relationships between recovered clades were not supported.

On ITS1 phylogeny four clades were identified: (1) *Cadlina* sp. 3 and *C. koltzovi*, (2) *C. laevis* HA and HB, (3) *C. umiushi* and *C. lomonosovi* and (4) *C. vinogradovi*. All recovered clades were highly supported and show no or little internal variation.

The ITS2 phylogeny recovered five clades: (1) *Cadlina* sp. 3 and *C. koltzovi*, (2) *C. laevis* HA, HB and *C. vavilovi*, (3) *C. umiushi* and *C. lomonosovi*, (4) *Cadlina* sp. 7 and (5) *Cadlina vinogradovi*. All recovered clades were highly supported and show no or little internal variation.

The concatenated dataset of all studied markers (COI, nDNA) provided a better-supported ML tree than the individual marker phylogenetic analyses (Figure S3). At the same time, the species grouping was different compared to that in the COI-based phylogenetic analysis (Figure 4). *Cadlina* sp. 3 was not recovered monophyletic and also contained *C. koltzovi* specimens as a derived internal subclade, although this group did not receive high support. Also, several species-level clades received moderate statistical support: *C. umiushi* (BS = 81), *C. lomonosovi* (BS = 87) and *C. laevis* (BS = 88); the monophyly of both *C. laevis* haplogroups was not supported.

The concatenated dataset of nDNA markers contained fewer specimens, as those with only 28S rRNA sequenced were excluded from the analysis due to a lack of phylogenetic resolution in 28S marker (Figure S2). The remaining samples formed three major clades on the ML tree (Figure S4): (1) *Cadlina laevis* HA and HB + *C. vavilovi*, (2) *Cadlina* sp. 7 and (3) *Cadlina* sp. 3 + *Cadlina koltzovi*; all these clades received moderate statistical support*. Cadlina umiushi* and *C. lomonosovi* formed an unresolved basal polytomy.

### 3.7. Divergence Times Estimation (DTE)

**Figure 8:**
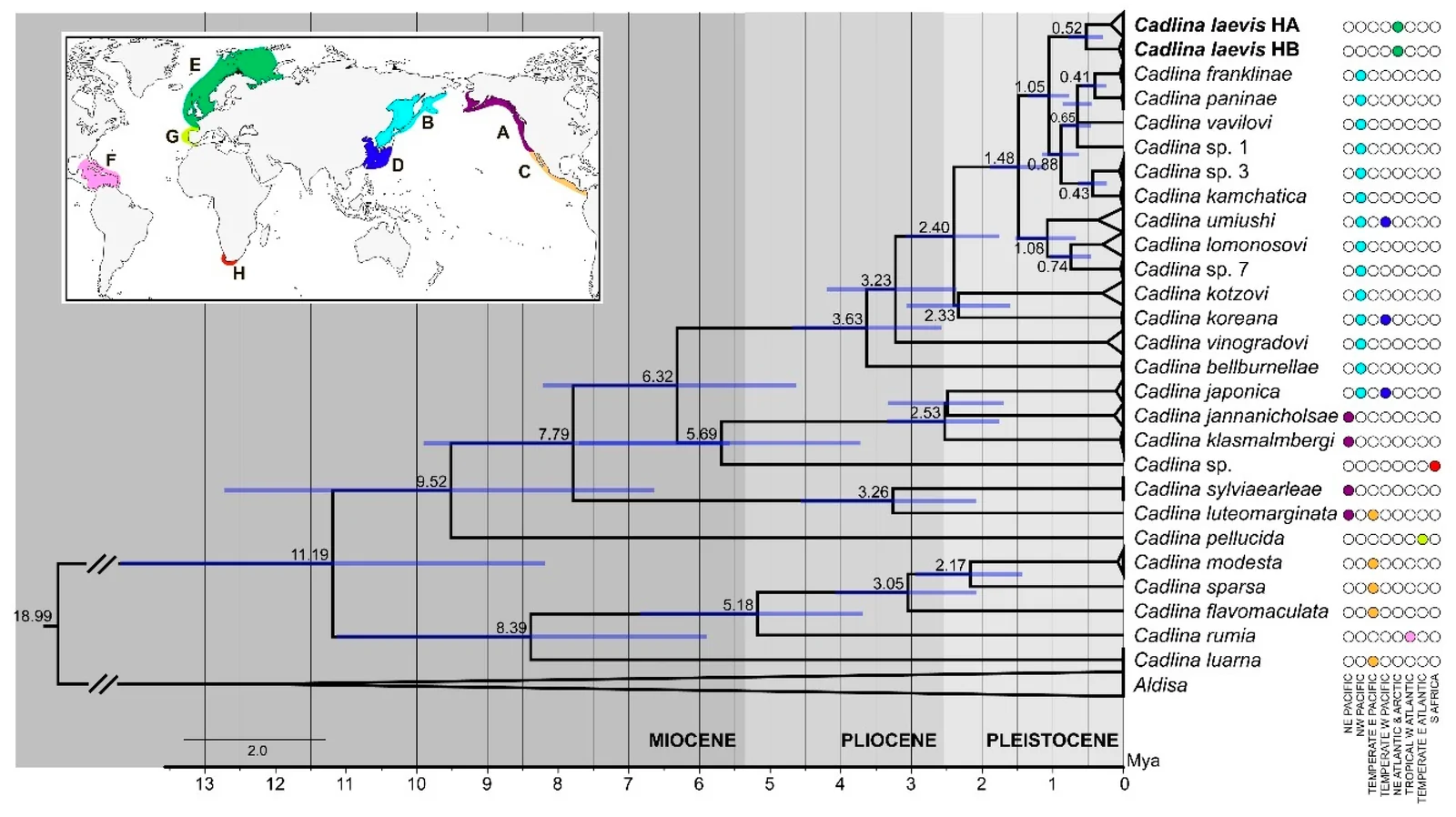
Chronogram of the genus *Cadlina*, based on the Birth–Death model from BEAST for the COI dataset using the tentative group-specific divergence rates of 6.1% per My for gastropods [46]. X-axis shows dating in Mya. Distribution ranges for each species are shown in blocks on the right and the map on the left shows the colour code for each region. HPD 95% intervals are shown for major nodes. A—boreal North-East Pacific. B—boreal North-West Pacific. C—temperate and subtropical North-East Pacific. D—temperate and subtropical North-West Pacific. E—North-East Atlantic and subarctic. F—Caribbean. G—temperate North-East Atlantic. H—South Africa.

The TMRCA of the *Cadlina laevis* species complex was dated at 3.63 Mya (±1.1 My) (Figure 8). The split into two haplogroups of the *Cadlina laevis* (HA and HB) was dated at 0.52 Mya (±150 Ky). This DTE corresponded to the MIS13 or Cromerian interglacial period between ~524,000 and 474,000 [51]. The DTE of *Cadlina laevis* HA and HB was higher than that of *C. franklinae* and *C. paninae* (0.41 Mya ± 100 Ky) and *C. kamchatica* and *Cadlina* sp. 3 (0.43 Mya ± 104 Ky). The DTE for interspecific splits between other *Cadlina* species from the North-East Pacific (except the *C. laevis* species complex) were dated at Pliocene/Pleistocene boundary in most cases, and therefore these estimates were much higher than those for most members within the *C. laevis* complex.

## 4. Discussion

### 4.1. mtDNA Variation in Cadlina laevis s.str.

The discovery of two divergent mitochondrial haplogroups (HA and HB) in the White Sea populations of *Cadlina laevis* s.str. represents a clear case of intraspecific mitochondrial diversity. The conserved gene order and synteny across all five compared *Cadlina* mitogenomes (Figure 2) indicate that the mitochondrial genome structure is highly stable within the genus, with no evidence of rearrangements.

Sliding-window analysis demonstrated that mitochondrial divergence was strongly associated with genomic position rather than being distributed uniformly across the mitogenome. Mean pairwise p-distance generally remained approximately within 0.05–0.15 across most regions but varied considerably between adjacent intervals. Relatively conserved regions were observed within the ribosomal RNA genes and several protein-coding regions, whereas higher divergence occurred within parts of the NADH dehydrogenase genes and the ATP synthase region. The most prominent local peak, approaching a mean p-distance of 0.30, was located at approximately 8.5–9.0 kb and coincided with the tRNA-rich interval adjacent to *ATP8* (Figure 2). The continuous divergence signal across the protein-coding portion of the genome demonstrates that the differentiation among the compared *Cadlina* mitogenomes is genome-wide rather than restricted to a single mitochondrial marker. This coordinate-dependent pattern was also consistent with the gene-by-gene analysis. Among the two White Sea *C. laevis* mitogenomes, p-distance ranged from 0.01037 in *COX2* to 0.05128 in *ATP6*, while *COX1* and *COX3* showed intermediate values of 0.02589 and 0.03333, respectively. The COI fragment used in the broader phylogenetic and species-delimitation analyses produced a wsbs1–wsbs2 p-distance of 0.02287, closely matching the value obtained for the complete *COX1* gene (Figure 3). This correspondence indicates that the standard COI fragment provides a representative estimate of *COX1* divergence between the two White Sea haplogroups and that their separation is not caused by an unusually divergent portion of the barcode region. At the same time, the substantially greater divergence observed in *ATP6* and several NADH dehydrogenase genes shows that COI captures only part of the total mitochondrial differentiation. Consequently, COI is informative for identifying the two mitochondrial lineages in the White Sea, but its divergence alone cannot be treated as a direct estimate of whole-mitogenome differentiation or as sufficient evidence of species-level separation. At the same time, variation in mtDNA length and relatively high p-distances between protein-coding genes (Figure 3) suggest that these two White Sea haplogroups have been isolated for a substantial evolutionary timescale.

Co-occurrence of two divergent mitochondrial haplogroups in the White Sea is particularly puzzling given the basin’s young geological history. The White Sea was formed only during the early Holocene marine transgression (approximately 10,000–12,000 years ago [12]), suggesting that either (1) both haplogroups arrived to the basin independently from different refugial populations during the post-glacial transgression, or (2) the divergence between these haplogroups occurred in the White Sea within a remarkably short timeframe. Our divergence times estimations dated the TMRCA of both haplogroups at MIS13 interglacial (0.52 Mya ± 150 Ky), a much older event than the White Sea formation, thus supporting the first scenario. However, the second scenario can also be a case of purely mitochondrial-haplogroup introgression with the resulting population only retaining nuclear genomes of one of the ancestors. But as the source for this putative haplogroup outside the White Sea is unknown, this is less plausible.

### 4.2. Mitonuclear Discordance in Cadlina laevis Species Complex

Disagreement in phylogenetic reconstructions derived from mitochondrial and nuclear data is a useful indicator of systematically problematic situations. Such cases directly challenge our interpretation of species identity in light of both the phylogenetic and biological species concepts. Even a limited number of markers was shown to disentangle evolutionary history and species identity with mitonuclear discordance [52,53]. For mollusks the nuclear non-coding ribosomal cluster elements have proved a reliable tool to screen for hybridization in closely related species with sensitivity on par with COI fragment traditionally used for DNA barcoding [54,55].

In our case phylogenetic reconstructions based on partial nuclear markers (18S, ITS1, ITS2) reveal a striking mitonuclear discordance in several *Cadlina* lineages that fundamentally challenges the species hypotheses derived from mitochondrial data alone (Figure 7). While the COI phylogeny (Figure 4) recovered 13 distinct clades and three singletons of *C. laevis* species complex with varying degrees of support, the nuclear phylogenies consistently collapsed several of these mitochondrial lineages into single, well-supported clades with minimal or no nucleotide variation.

The two White Sea haplogroups (HA and HB) formed a single, highly supported clade in all nuclear phylogenies, with no detectable genetic differentiation across the nuclear markers examined, except for ITS1. This lack of nuclear genetic structure between the two mitochondrial haplogroups suggests some level of ongoing gene flow in autosomal genes or recent shared ancestry. No evidence on morphological or ecological differentiation of these haplogroups has been identified [11], implying they likely represent a single species-level taxonomical unit.

Such discordance extends beyond the White Sea populations of *Cadlina* species. Our nuclear phylogenies repeatedly grouped several mitochondrial clades that were considered distinct species in recent works. Among them, *C. laevis* HA, HB, and *C. vavilovi* formed a single clade in both 18S and ITS2 analyses (ITS1 amplification was not successful for *C. vavilovi*). In contrast to *C. laevis*, *Cadlina vavilovi* is the North-West Pacific species, and the lack of divergence in fast-evolving ITS2 is unexpected as these two species are not sister in both COI-based phylogenies (Figure 4 and Figure S1). The same pattern was observed for two other North-West Pacific species *Cadlina koltzovi* (the Kurile Is., the Sea of Japan) and *Cadlina* sp. 3 (the Sea of Japan). *Cadlina koltzovi* has a highly derived COI from other species of the complex (Figure 4, Figure 6 and Figure 7), while *Cadlina* sp. 3 represents sister relationships to *C. kamchatica* (NW Pacific, off Kamchatka). In nDNA analyses *C. koltzovi* and *Cadlina* sp. 3 were consistently grouped together across all nuclear markers, despite being separated by substantial COI p-distances. Also, nDNA phylogenetic analyses grouped together *Cadlina umiushi* (the Sea of Japan) and *C. lomonosovi* (the Kurile Islands).

These patterns of mitonuclear discordance can be attributed to several non-mutually exclusive evolutionary processes. First, incomplete lineage sorting may be a plausible explanation, particularly given the relatively recent diversification of the *Cadlina laevis* species complex (Figure 8). Histone H3 and nuclear rRNAs usually show little variation across closely related species [56]. However, ITS1 and ITS2 commonly provide a good resolution at low taxonomical levels in nudibranchs [57,58,59]. The conspecificity in these markers may indicate a contemporary autosomal gene flow between different mitochondrial clades rather than incomplete allele sorting. The case with the White Sea haplogroups is thus similar to the recently documented pattern in the *Zelentia pustulata* (Alder & Hancock, 1854) species complex [15]. Under this scenario, the two White Sea haplogroups of *C. laevis* would represent the descendants of different glacial refugial populations that colonized the newly formed White Sea basin in Holocene from separate source areas (e.g., the North Atlantic and the North Pacific, or different refugia within the Barents–Kara shelf). Upon secondary contact in the White Sea, they began to admix, but the mitochondrial lineages have not yet equilibrated due to their maternal inheritance, smaller effective population size, and possibly selection, while nuclear genes have been homogenized by rapid and unrestricted gene flow.

At the same time, no variation in nDNA markers between the Atlantic species *C. laevis* and the Pacific species *C. vavilovi* (Figure 7) was unexpected. Although recent studies identified numerous cases of possible recent geneflow in trans-Arctic allopatric populations in nudibranchs (e.g., in *Coryphella verrucosa* (M. Sars, 1829), *Onchidoris muricata* (O. F. Müller, 1776) and *Diaphoreolis viridis* (Forbes, 1840), see [14,59,60]), it is suggested that the gene flow may be facilitated by the dispersal potential of planktotrophic larvae. However, in the case of the *Cadlina laevis* species complex this possibility is in strong contradiction with data on their life cycle. Developmental studies showed the lack of free-living veligers at least in the Atlantic species *Cadlina laevis* [61]. This direct development mode dramatically reduces dispersal capacity, making contemporary trans-Arctic gene flow highly unlikely under current oceanographic conditions. Unfortunately, our material lacks specimens of other closely related species to *Cadlina vavilovi*: *C. paninae* and *C. franklinae*, for which nDNA data might provide additional lines of evidence either for or against possible hybridization.

While the scenarios of historical admixture and incomplete lineage sorting provide a parsimonious explanation for the observed discordance, alternative hypotheses merit consideration. Nuclear mitochondrial pseudogenes (NUMTs) could, in principle, produce artifactual mtDNA phylogenies [62,63]. However, the absence of stop codons and frameshifts in our COI alignments, along with the consistent signal across multiple mtDNA markers at least in the case of the species studied herein (Figure 2), makes widespread NUMTs contamination unlikely. Similarly, a replacement of the mitochondrial genome of one lineage by that of another through hybridization and mitochondrial introgression could generate discordance without nuclear homogenization [5,6], but this process alone would not explain the complete lack of nuclear differentiation between many mitochondrial clades across vast geographic distances. Finally, recent population bottlenecks and expansions could also contribute to the observed patterns. Disentangling these non-mutually exclusive processes will require genome-wide data screening, but the recurrent mitonuclear discordance in the *C. laevis* complex, coupled with morphological stasis and biogeographic context, strongly suggests that mitochondrial divergence is an unreliable proxy for species boundaries in this group.

### 4.3. Taxonomical Implication Within Cadlina laevis Species Complex

Apparent failure of species-delimitation analyses to provide consistent results further underscores the challenges of relying on mitochondrial data alone. The ASAP analyses united *C. laevis* HA and HB into a single operational unit, but also grouped together *Cadlina umiushi* and *Cadlina* sp. 7, which is inconsistent with our nDNA findings (Figure 4 and Figure 7). The PTP analyses collapsed mitochondrial clades of even broader range (including *C. franklinae*, *C. paninae*, *Cadlina* sp. 1, *C. vavilovi*, *Cadlina* sp. 3, *C. kamchatica*, and both *C. laevis* haplogroups) into a single unit. More importantly, at least *Cadlina laevis* and *C. vavilovi* showed no variation in nDNA markers, thus partly supporting the PTP results (Figure 7).

The COI-wide p-distance distribution (Figure 5) provides additional context for interpreting these patterns. The presence of overlapping intra- and interspecific distance ranges, visualized in the heatmap (Figure 6), indicates that using a universal distance threshold for species delimitation in *Cadlina* is unlikely to be successful. The 2.5% COI divergence between the White Sea haplogroups HA and HB falls within the “grey zone” of ambiguous species boundaries, where both intraspecific (*Cadlina laevis*, *C. lomonosovi* and *C. umiushi* haplogroups) and interspecific (i.e., *Cadlina paninae* vs. *C. franklinae*) interpretations are equally plausible. Crucially, most delimitation methods assume strict bifurcating speciation without gene flow and perform poorly when lineages experienced temporary isolation followed by secondary contact—a common scenario in high-latitude taxa, as recently shown for *Zelentia pustulata* [15] and *Priapulus caudatus* Lamarck, 1816 [64].

Results of this study have profound implications for the taxonomy of the *Cadlina laevis* species complex and raise important questions about the viability of the current species-level taxonomy. Our extensive evidence—particularly the strong mitonuclear discordance and the lack of consistent morphological differences between mitochondrial clades—suggests that the hypothesis of more than ten separate species proposed by [10] may represent an over-splitting of mitochondrial lineages. Previous studies of this complex provided a vast morphological study of all clades discussed herein [10,11,29]. Despite extensive examinations, no consistent morphological characters have been found to differentiate the mitochondrial clades [10,11,29]. Overall, several species may have yellowish (e.g., *C. kamchatica*) or white coloration (e.g., *C. paninae*) of both colour morphs (e.g., *C. laevis*), different number of denticles on the rachidian teeth of radula, different length of vas deferens in the reproductive system and other fine features. Korshunova & Martynov [10] reported some “fine-scale morphological features” that may overlap even in non-closely related species, e.g., *C. laevis* and *C. vavilovi* [10], or *C. umiushi* and *C. vinogradovi* (as *Cadlina* sp. in [29]). The existence of such subtle, non-diagnostic differences is inconsistent with the recognition of distinct species. Thus, the available evidence does not support a one-to-one correspondence between the currently recognized mitochondrial lineages and independently evolving species. Further taxonomic revision of *Cadlina laevis* species complex therefore requires geographically representative genome-wide nuclear and phenotypic data.

## 5. Conclusions

The pervasive mitonuclear discordance documented here confirms that COI barcodes alone are inadequate for species delimitation in the *Cadlina laevis* complex. To understand why mitochondrial markers perform so poorly, further studies would benefit from a geographically comprehensive dataset of complete mitogenomes from under-represented North Pacific lineages, and wider array of autosomal markers. Comparative mitochondrial genomic data will reveal whether the observed high polymorphism and gene specific rate heterogeneity are widespread or lineage-specific. Together with genome-wide nuclear and phenotypic evidence, this mitogenomic framework is essential to disentangle the evolutionary drivers of mitochondrial diversity and to build a stable, biologically meaningful taxonomy for the group in further studies.

## Data Availability

All newly obtained partial sequences and full mitochondrial genomes were submitted to GenBank database. All custom R scripts used in this study and unedited output files of species-delimitation analyses are uploaded as Supplementary Materials.

## Supplementary Materials

The following supporting information can be downloaded at https://www.mdpi.com/article/10.3390/ani16182934/s1, Figure S1: Bayesian phylogenetic tree of the genus *Cadlina* based on the COI dataset, numbers in nodes indicate posterior probabilities; Figure S2: Maximum-likelihood trees of the *Cadlina laevis* species complex based on partial histone H3 and 28S rRNA datasets, numbers in nodes indicate respective bootstrap support; Figure S3: Maximum-likelihood tree of the *Cadlina laevis* species complex, based on the concatenated dataset of COI and all available nuclear markers. Only species with at least one sequenced nuclear marker are included in the phylogeny. Numbers in nodes indicate respective bootstrap support; Figure S4: Maximum-likelihood tree of the *Cadlina laevis* species complex, based on the concatenated dataset of nDNA markers. Only specimens with at least two sequenced nuclear markers are included in the phylogeny. Numbers in nodes indicate respective bootstrap support; Table S1: List of specimens used in this study with respective voucher number, DNA number, collection locality and GB accession number for each sample; Table S2: Amplification and sequencing primers with respective PCR conditions and best model for phylogenetic analysis; Table S3: Summary of pairwise comparisons of partial COI dataset; Data S1: Custom R scripts used in this study; Data S2: Unedited output files from species-delimitation analyses.

## References

[1] Carstens B.C., Pelletier T.A., Reid N.M., Satler J.D. (2013). How to fail at species delimitation. Mol. Ecol..

[2] Dellicour S., Flot J.F. (2018). The hitchhiker’s guide to single-locus species delimitation. Mol. Ecol. Resour..

[3] Doellman M.M., Trussell G.C., Grahame J.W., Vollmer S.V. (2011). Phylogeographic analysis reveals a deep lineage split within North Atlantic *Littorina saxatilis*. Proc. R. Soc. B.

[4] Ekimova I.A., Mikhlina A.L., Vorobyeva O.A., Antokhina T.I., Tambovtseva V.G., Schepetov D.M. (2021). Young but distinct: Description of *Eubranchus malakhovi* sp. n. a new, recently diverged nudibranch species (Gastropoda: Heterobranchia) from the Sea of Japan. Invertebr. Zool..

[5] Yuan Z., Wu D., Wen Y., Xu W., Gao W., Dahn H.A., Liu X., Jin J., Yu C., Xiao H. (2023). Historical mitochondrial genome introgression confounds species delimitation: Evidence from phylogenetic inference in the *Odorrana grahami* species complex. Curr. Zool..

[6] Thielsch A., Knell A., Mohammadyari A., Petrusek A., Schwenk K. (2017). Divergent clades or cryptic species? Mito-nuclear discordance in a *Daphnia* species complex. BMC Evol. Biol..

[7] Weber A.A.T., Stöhr S., Chenuil A. (2019). Species delimitation in the presence of strong incomplete lineage sorting and hybridization: Lessons from *Ophioderma* (Ophiuroidea: Echinodermata). Mol. Phylogenet. Evol..

[8] Toews D.P., Brelsford A. (2012). The biogeography of mitochondrial and nuclear discordance in animals. Mol. Ecol..

[9] Korshunova T., Fletcher K., Picton B., Lundin K., Kashio S., Sanamyan N., Padula V., Schrodl M., Martynov A. (2020). The Emperor’s *Cadlina*, hidden diversity and gill cavity evolution: New insights for the taxonomy and phylogeny of dorid nudibranchs (Mollusca: Gastropoda). Zool. J. Linn. Soc..

[10] Korshunova T., Martynov A. (2024). The phyloperiodic approach removes the “cryptic species” and puts forward multilevel organismal diversity. Diversity.

[11] Ekimova I.A., Grishina D.Y., Valdés Á., Antokhina T.I., Chichvarkhina O.V., Schepetov D.M. (2024). Fifty shades of white: Morphological and molecular diversity of the *Cadlina laevis* species complex (Gastropoda: Nudibranchia) in the North-West Pacific. Ruthenica.

[12] Korsakova O. (2026). Late Pleistocene–Early Holocene depositional records from proglacial and periglacial basins in the north-eastern Europe: Chronological correlation across the White Sea depression. Quat. Int..

[13] Borovikova E.A., Malina J.I. (2018). Phylogeography of common whitefish (*Coregonus lavaretus* L.) of Northwestern Russia. Contemp. Probl. Ecol..

[14] Ekimova I.A., Nikitenko E., Stanovova M.V., Schepetov D.M., Antokhina T.I., Malaquias M.A.E., Valdés Á. (2023). Unity in diversity: Morphological and genetic variability, integrative systematics, and phylogeography of the widespread nudibranch mollusc *Onchidoris muricata*. Syst. Biodivers..

[15] Indriksone J.V., Schepetov D.M., Tambovtseva V.G., Antokhina T.I., Ekimova I.A. (2026). Integrative systematics and phylogeographical patterns suggest the *Zelentia pustulata* species complex (Gastropoda: Nudibranchia) represents a single species with a complex evolutionary history. Zool. Anz..

[16] Ivanova N.V., Dewaard J.R., Hebert P.D. (2006). An inexpensive, automation-friendly protocol for recovering high-quality DNA. Mol. Ecol. Notes.

[17] Ekimova I.A., Grishina D.Y., Nikitenko E.D. (2024). Nudibranch molluscs of Sakhalin Island, Northwestern Pacific: New records and descriptions of two new species. Ruthenica.

[18] Prjibelski A., Antipov D., Meleshko D., Lapidus A., Korobeynikov A. (2020). Using SPAdes de novo assembler. Curr. Protoc. Bioinform..

[19] Bernt M., Donath A., Jühling F., Externbrink F., Florentz C., Fritzsch G., Putz J., Middendorf M., Stadler P.F. (2013). MITOS: Improved de novo metazoan mitochondrial genome annotation. Mol. Phylogenet. Evol..

[20] Langmead B., Salzberg S.L. (2012). Fast gapped-read alignment with Bowtie 2. Nat. Methods.

[21] Li H., Handsaker B., Wysoker A., Fennell T., Ruan J., Homer N., Marth G., Abecasis G., Durbin R. (2009). 1000 Genome Project Data Processing Subgroup. The sequence alignment/map format and SAMtools. Bioinformatics.

[22] Gu Z., Gu L., Eils R., Schlesner M., Brors B. (2014). Circlize implements and enhances circular visualization in R. Bioinformatics.

[23] Do T.D., Jung D.W., Kim C.B. (2022). Molecular phylogeny of selected dorid nudibranchs based on complete mitochondrial genome. Sci. Rep..

[24] Hackl T., Ankenbrand M., van Adrichem B. (2024). gggenomes: A Grammar of Graphics for Comparative Genomics.

[25] Ranwez V., Douzery E.J., Cambon C., Chantret N., Delsuc F. (2018). MACSE v2: Toolkit for the alignment of coding sequences accounting for frameshifts and stop codons. Mol. Biol. Evol..

[26] Lemoine F., Gascuel O. (2021). Gotree/Goalign: Toolkit and Go API to facilitate the development of phylogenetic workflows. NAR Genom. Bioinform..

[27] Wickham H., Averick M., Bryan J., Chang W., McGowan L.D.A., François R., Grolemund G., Hayes A., Henry L., Hester J. (2019). Welcome to the Tidyverse. J. Open Source Softw..

[28] Wickham H. (2016). Data analysis. ggplot2: Elegant Graphics for Data Analysis.

[29] Ekimova I., Valdés Á., Stanovova M., Mikhlina A., Antokhina T., Neretina T., Chichvarkhina O., Schepetov D. (2021). Connected across the ocean: Taxonomy and biogeography of deep-water Nudibranchia from the Northwest Pacific reveal trans-Pacific links and two undescribed species. Org. Divers. Evol..

[30] Edgar R.C. (2004). MUSCLE: Multiple sequence alignment with high accuracy and high throughput. Nucleic Acids Res..

[31] Kumar S., Stecher G., Tamura K. (2016). MEGA7: Molecular evolutionary genetics analysis version 7.0 for bigger datasets. Mol. Biol. Evol..

[32] Ronquist F., Teslenko M., Van Der Mark P., Ayres D.L., Darling A., Höhna S., Larget B., Liu L., Suchard M.A., Huelsenbeck J.P. (2012). MrBayes 3.2: Efficient Bayesian phylogenetic inference and model choice across a large model space. Syst. Biol..

[33] Edler D., Klein J., Antonelli A., Silvestro D. (2021). raxmlGUI 2.0: A graphical interface and toolkit for phylogenetic analyses using RAxML. Methods Ecol. Evol..

[34] Pattengale N.D., Alipour M., Bininda-Emonds O.R., Moret B.M., Stamatakis A. (2010). How many bootstrap replicates are necessary?. J. Comput. Biol..

[35] Rambaut A. (2010). FigTree.

[36] Adobe Inc. (2019). Adobe Illustrator.

[37] Chaban E.M., Ekimova I.A., Schepetov D.M., Chernyshev A.V. (2019). Meloscaphander grandis (Heterobranchia: Cephalaspidea), a deep-water species from the North Pacific: Redescription and taxonomic remarks. Zootaxa.

[38] Stamatakis A. (2014). RAxML version 8: A tool for phylogenetic analysis and post-analysis of large phylogenies. Bioinformatics.

[39] Stamatakis A., Hoover P., Rougemont J. (2008). A rapid bootstrap algorithm for the RAxML web servers. Syst. Biol..

[40] Puillandre N., Brouillet S., Achaz G. (2021). ASAP: Assemble species by automatic partitioning. Mol. Ecol. Resour..

[41] Vences M., Miralles A., Brouillet S., Ducasse J., Fedosov A., Kharchev V., Kostadinov I., Kumari S., Patmanidis S., Scherz M.D. (2021). iTaxoTools 0.1: Kickstarting a specimen-based software toolkit for taxonomists. Megataxa.

[42] Zhang J., Kapli P., Pavlidis P., Stamatakis A. (2013). A general species delimitation method with applications to phylogenetic placements. Bioinformatics.

[43] Pons J., Barraclough T.G., Gomez-Zurita J., Cardoso A., Duran D.P., Hazell S., Kamoun S., Sumlin W.D., Vogler A.P. (2006). Sequence-based species delimitation for the DNA taxonomy of undescribed insects. Syst. Biol..

[44] Fujisawa T., Barraclough T.G. (2013). Delimiting species using single-locus data and the Generalized Mixed Yule Coalescent approach: A revised method and evaluation on simulated data sets. Syst. Biol..

[45] Bouckaert R., Heled J., Kühnert D., Vaughan T., Wu C.H., Xie D., Suchard M.A., Rambaut A., Drummond A.J. (2014). BEAST 2: A software platform for Bayesian evolutionary analysis. PLoS Comput. Biol..

[46] Laakkonen H.M., Hardman M., Strelkov P., Väinölä R. (2021). Cycles of trans-Arctic dispersal and vicariance, and diversification of the amphi-boreal marine fauna. J. Evol. Biol..

[47] Rambaut A., Drummond A.J., Xie D., Baele G., Suchard M.A. (2018). Posterior summarization in Bayesian phylogenetics using Tracer 1.7. Syst. Biol..

[48] Hennig W. (1966). Phylogenetic Systematics.

[49] Wiley E.O. (1978). The Evolutionary Species Concept Reconsidered. Syst. Zool..

[50] Moles J., Bonomo L., Brenzinger B., Camacho García Y.E., Carmona L., Cervera Currado J.L., Donohoo S., Fernández-Simón J., Furfaro G., Galià-Camps C. (2026). Towards systematics-based guidelines for stable taxonomy: Lessons from sea slugs. Zool. J. Linn. Soc..

[51] Lisiecki L.E., Raymo M.E. (2005). A Pliocene-Pleistocene stack of 57 globally distributed benthic δ18O records. Paleoceanography.

[52] Hernandez J., Cognato A.I. (2024). Incomplete barriers to heterospecific mating among Somatochlora species (Odonata: Corduliidae) as revealed in multi-gene phylogenies. Cladistics.

[53] Sousa-Santos C., Pereira A.M., Branco P., Costa G.J., Santos J.M., Ferreira M.T., Lima C.S., Doadrio I., Robalo J.I. (2018). Mito-nuclear sequencing is paramount to correctly identify sympatric hybridizing fishes. Acta Ichthyol. Piscat..

[54] Salvi D., Macali A., Mariottini P. (2014). Molecular phylogenetics and systematics of the bivalve family Ostreidae based on rRNA sequence-structure models and multilocus species tree. PLoS ONE.

[55] Catanese G., Vázquez-Luis M., Giacobbe S., García-March J.R., Zotou M., Patricia P., Papadakis O., Tena-Medialdea J., Katsanevakis S., Grau A. (2024). Internal transcribed spacer as effective molecular marker for the detection of natural hybridization between the bivalves *Pinna nobilis* and *Pinna rudis*. Ecol. Evol..

[56] Ekimova I., Valdes A., Malaquias M.A.E., Rauch C., Chichvarkhin A., Mikhlina A., Antokhina T., Chichvakhina O., Schepetov D. (2022). High-level taxonomic splitting in allopatric taxa causes confusion downstream: A revision of the nudibranch family Coryphellidae. Zool. J. Linn. Soc..

[57] Salvi D., Mariottini P. (2017). Molecular taxonomy in 2D: A novel ITS2 rRNA sequence-structure approach guides the description of the oysters’ subfamily Saccostreinae and the genus *Magallana* (Bivalvia: Ostreidae). Zool. J. Linn. Soc..

[58] Garzia M., Mariottini P., Salvi D., Furfaro G. (2021). Variation and diagnostic power of the internal transcribed spacer 2 in Mediterranean and Atlantic eolid nudibranchs (Mollusca, Gastropoda). Front. Mar. Sci..

[59] Grishina D., Schepetov D., Mikhlina A., Antokhina T., Deart Y., Ekimova I. (2026). What You Can See from here: A Critical Role of Integrative Approach and Sample Size in Defining Species Boundaries for Trans-Arctic Nudibranchs (Gastropoda: Heterobranchia). Zool. Scr..

[60] Ekimova I.A., Schepetov D.M., Green B., Stanovova M.V., Antokhina T.I., Gosliner T., Malaquias M.A.E., Valdés Á. (2024). Scaling the high latitudes: Evolution, diversification, and dispersal of *Coryphella* nudibranchs across the Northern Hemisphere. Mol. Phylogenet. Evol..

[61] Thompson T.E. (1967). Direct development in a nudibranch, *Cadlina laevis*, with a discussion of developmental processes in Opisthobranchia. J. Mar. Biol. Assoc. UK.

[62] Song H., Buhay J.E., Whiting M.F., Crandall K.A. (2008). Many species in one: DNA barcoding overestimates the number of species when nuclear mitochondrial pseudogenes are coamplified. Proc. Natl. Acad. Sci. USA.

[63] Liu H., Liu Y., Hu Y. (2025). Mitochondrial Impostors: Prevalence and Impacts of NUMTs on Genetic and Evolutionary Studies in Carnivora. Genome Biol. Evol..

[64] Kolbasova G., Schmidt-Rhaesa A., Syomin V., Bredikhin D., Morozov T., Neretina T. (2023). Cryptic species complex or an incomplete speciation? Phylogeographic analysis reveals an intricate Pleistocene history of *Priapulus caudatus* Lamarck, 1816. Zool. Anz..

