# Supplementary material for: High Intraspecific mtDNA Polymorphisms and Mitonuclear Discordance: A Case Study of the Cadlina laevis (Linnaeus, 1767) Species Complex
Source: Animals (Basel). 2026 Sep 18;16(18):2934. doi: 10.3390/ani16182934 (PMC13603891; doi:10.3390/ani16182934)
Supplement: Supplementary file 1 [file animals-16-02934-s001.zip › animals-4512781-supplementary/Table S2.pdf]

Table S2. Amplification and sequencing primers and PCR conditions, and evolutionary models for respective partitions in phylogenetic analyses.

Marker	Primers	PCR conditions	References	Evolutionary model
Cytochrome *c* oxidase subunit I (COI)	**LCO1490** GGTCAACAAATCATAAAGATATTGG **HCO2198** TAAACTTCAGGGTGACCAAAAAATCA	5 min – 95 °C, 35x [15 s – 95 °C, 30 s – 45 °C, 1 min – 72 °C], 7 min – 72 °C	Folmer et al., 1994	GTR+G+I
Histone H3	**H3AF** ATGGCTCGTACCAAGCAGACVGC **H3AR** ATATCCTTRGGCATRATRGTGAC	5 min – 95 °C, 35x [15 s – 95 °C, 30 s – 50 °C, 1 min – 72 °C], 7 min – 72 °C	Colgan et al., 1998	K2P+G
18S rRNA	**1F** TACCTGGTTGATCCTGCCAGTAG **5R** CTTGGCAAATGCTTTCGC **3F** GTTCGATTCCGGAGAGGGA **18Sbi** GAGTCTCGTTCGTTATCGGA **18Sa2.0** ATGGTTGCAAAGCTGAAAC **9R** GATCCTTCCGCAGGTTCACCTAC	5 min – 95 °C, 35x [30 s – 95 °C, 30 s – 50 °C, 1 min – 72 °C], 7 min – 72 °C	Giribet et al., 1996; Whiting et al., 1997	K2P
ITS1	**NudITS1_F** GTAAAAGTCGTAACAAGGTT **NudITS1_R** TTTCACACGAYTTCMCGCGGC	5 min – 95 °C, 5x [20 s – 95 °C, 40 s – 47 °C, -0,5 °C, 1 min – 72 °C], 35x [20 s – 95 °C, 30 s – 45 °C, 40 s – 72 °C], 7 min – 72 °C	Indriksone et al., 2026	HKY
ITS2	**NudITS2_F** CGGATCACTCGGCTCGCGC **NudITS2 R** AGTTTYTTTTCCTCCGCTTA	5 min – 95 °C, 5x [20 s – 95 °C, 40 s – 47 °C, -0,5 °C, 1 min – 72 °C], 35x [20 s – 95 °C, 30 s – 45 °C, 40 s – 72 °C], 7 min – 72 °C	Indriksone et al., 2026	HKY+G
