# Supplementary material for: High Intraspecific mtDNA Polymorphisms and Mitonuclear Discordance: A Case Study of the Cadlina laevis (Linnaeus, 1767) Species Complex
Source: Animals (Basel). 2026 Sep 18;16(18):2934. doi: 10.3390/ani16182934 (PMC13603891; doi:10.3390/ani16182934)
Supplement: Supplementary file 1 [file animals-16-02934-s001.zip › animals-4512781-supplementary/Data S2.zip!/Cadlin_COI_GMYC.pdf]

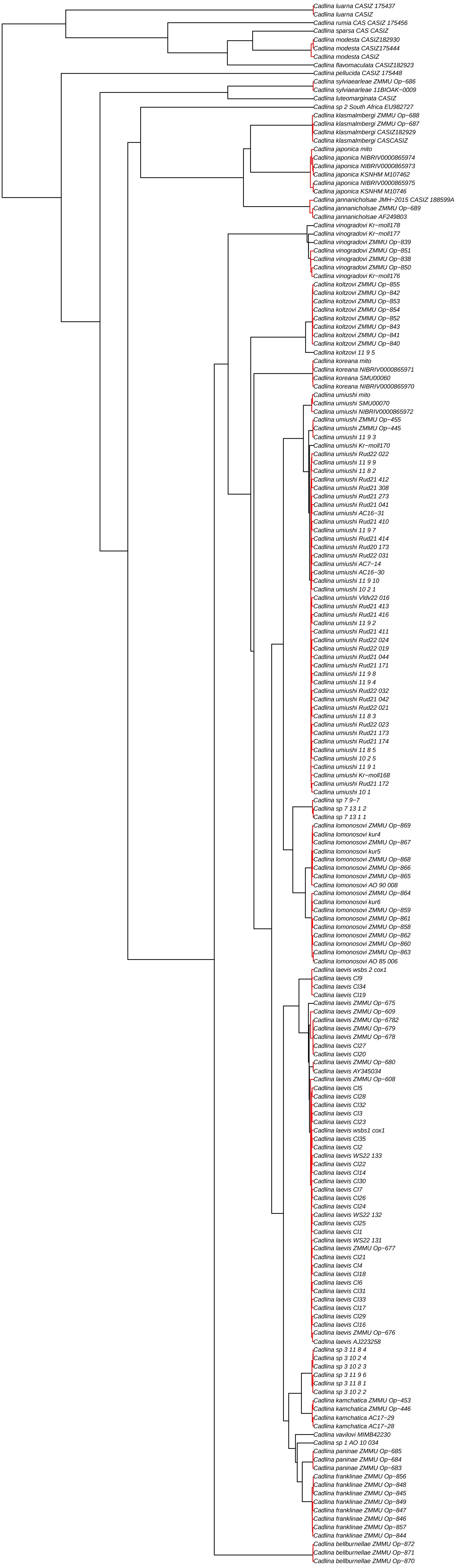
Cadlina luarna CASIZ 175437
Cadlina luarna CASIZ
Cadlina rumia CAS CASIZ 175456
Cadlina sparsa CAS CASIZ
Cadlina modesta CASIZ182930
Cadlina modesta CASIZ175444
Cadlina modesta CASIZ
Cadlina flavomaculata CASIZ182923
Cadlina pellucida CASIZ 175448
Cadlina sylviaearleae ZMMU Op−686
Cadlina sylviaearleae 11BIOAK−0009
Cadlina luteomarginata CASIZ
Cadlina sp 2 South Africa EU982727
Cadlina klasmalmbergi ZMMU Op−688
Cadlina klasmalmbergi ZMMU Op−687
Cadlina klasmalmbergi CASIZ182929
Cadlina klasmalmbergi CASCASIZ
Cadlina japonica mito
Cadlina japonica NIBRIV0000865974
Cadlina japonica NIBRIV0000865973
Cadlina japonica KSNHM M107462
Cadlina japonica NIBRIV0000865975
Cadlina japonica KSNHM M10746
Cadlina jannanicholsae JMH−2015 CASIZ 188599A
Cadlina jannanicholsae ZMMU Op−689
Cadlina jannanicholsae AF249803
Cadlina vinogradovi Kr−moll178
Cadlina vinogradovi Kr−moll177
Cadlina vinogradovi ZMMU Op−839
Cadlina vinogradovi ZMMU Op−851
Cadlina vinogradovi ZMMU Op−838
Cadlina vinogradovi ZMMU Op−850
Cadlina vinogradovi Kr−moll176
Cadlina koltzovi ZMMU Op−855
Cadlina koltzovi ZMMU Op−842
Cadlina koltzovi ZMMU Op−853
Cadlina koltzovi ZMMU Op−854
Cadlina koltzovi ZMMU Op−852
Cadlina koltzovi ZMMU Op−843
Cadlina koltzovi ZMMU Op−841
Cadlina koltzovi ZMMU Op−840
Cadlina koltzovi 11 9 5
Cadlina koreana mito
Cadlina koreana NIBRIV0000865971
Cadlina koreana SMU00060
Cadlina koreana NIBRIV0000865970
Cadlina umiushi mito
Cadlina umiushi SMU00070
Cadlina umiushi NIBRIV0000865972
Cadlina umiushi ZMMU Op−455
Cadlina umiushi ZMMU Op−445
Cadlina umiushi 11 9 3
Cadlina umiushi Kr−moll170
Cadlina umiushi Rud22 022
Cadlina umiushi 11 9 9
Cadlina umiushi 11 8 2
Cadlina umiushi Rud21 412
Cadlina umiushi Rud21 308
Cadlina umiushi Rud21 273
Cadlina umiushi Rud21 041
Cadlina umiushi AC16−31
Cadlina umiushi Rud21 410
Cadlina umiushi 11 9 7
Cadlina umiushi Rud21 414
Cadlina umiushi Rud20 173
Cadlina umiushi Rud22 031
Cadlina umiushi AC7−14
Cadlina umiushi AC16−30
Cadlina umiushi 11 9 10
Cadlina umiushi 10 2 1
Cadlina umiushi Vldv22 016
Cadlina umiushi Rud21 413
Cadlina umiushi Rud21 416
Cadlina umiushi 11 9 2
Cadlina umiushi Rud21 411
Cadlina umiushi Rud22 024
Cadlina umiushi Rud22 019
Cadlina umiushi Rud21 044
Cadlina umiushi Rud21 171
Cadlina umiushi 11 9 8
Cadlina umiushi 11 9 4
Cadlina umiushi Rud22 032
Cadlina umiushi Rud21 042
Cadlina umiushi Rud22 021
Cadlina umiushi 11 8 3
Cadlina umiushi Rud22 023
Cadlina umiushi Rud21 173
Cadlina umiushi Rud21 174
Cadlina umiushi 11 8 5
Cadlina umiushi 10 2 5
Cadlina umiushi 11 9 1
Cadlina umiushi Kr−moll168
Cadlina umiushi Rud21 172
Cadlina umiushi 10 1
Cadlina sp 7 9−7
Cadlina sp 7 13 1 2
Cadlina sp 7 13 1 1
Cadlina lomonosovi ZMMU Op−869
Cadlina lomonosovi kur4
Cadlina lomonosovi ZMMU Op−867
Cadlina lomonosovi kur5
Cadlina lomonosovi ZMMU Op−868
Cadlina lomonosovi ZMMU Op−866
Cadlina lomonosovi ZMMU Op−865
Cadlina lomonosovi AO 90 008
Cadlina lomonosovi ZMMU Op−864
Cadlina lomonosovi kur6
Cadlina lomonosovi ZMMU Op−859
Cadlina lomonosovi ZMMU Op−861
Cadlina lomonosovi ZMMU Op−858
Cadlina lomonosovi ZMMU Op−862
Cadlina lomonosovi ZMMU Op−860
Cadlina lomonosovi ZMMU Op−863
Cadlina lomonosovi AO 85 006
Cadlina laevis wsbs 2 cox1
Cadlina laevis Cl9
Cadlina laevis Cl34
Cadlina laevis Cl19
Cadlina laevis ZMMU Op−675
Cadlina laevis ZMMU Op−609
Cadlina laevis ZMMU Op−6782
Cadlina laevis ZMMU Op−679
Cadlina laevis ZMMU Op−678
Cadlina laevis Cl27
Cadlina laevis Cl20
Cadlina laevis ZMMU Op−680
Cadlina laevis AY345034
Cadlina laevis ZMMU Op−608
Cadlina laevis Cl5
Cadlina laevis Cl28
Cadlina laevis Cl32
Cadlina laevis Cl3
Cadlina laevis Cl23
Cadlina laevis wsbs1 cox1
Cadlina laevis Cl35
Cadlina laevis Cl2
Cadlina laevis WS22 133
Cadlina laevis Cl22
Cadlina laevis Cl14
Cadlina laevis Cl30
Cadlina laevis Cl7
Cadlina laevis Cl26
Cadlina laevis Cl24
Cadlina laevis WS22 132
Cadlina laevis Cl25
Cadlina laevis Cl1
Cadlina laevis WS22 131
Cadlina laevis ZMMU Op−677
Cadlina laevis Cl21
Cadlina laevis Cl4
Cadlina laevis Cl18
Cadlina laevis Cl6
Cadlina laevis Cl31
Cadlina laevis Cl33
Cadlina laevis Cl17
Cadlina laevis Cl29
Cadlina laevis Cl16
Cadlina laevis ZMMU Op−676
Cadlina laevis AJ223258
Cadlina sp 3 11 8 4
Cadlina sp 3 10 2 4
Cadlina sp 3 10 2 3
Cadlina sp 3 11 9 6
Cadlina sp 3 11 8 1
Cadlina sp 3 10 2 2
Cadlina kamchatica ZMMU Op−453
Cadlina kamchatica ZMMU Op−446
Cadlina kamchatica AC17−29
Cadlina kamchatica AC17−28
Cadlina vavilovi MIMB42230
Cadlina sp 1 AO 10 034
Cadlina paninae ZMMU Op−685
Cadlina paninae ZMMU Op−684
Cadlina paninae ZMMU Op−683
Cadlina franklinae ZMMU Op−856
Cadlina franklinae ZMMU Op−848
Cadlina franklinae ZMMU Op−845
Cadlina franklinae ZMMU Op−849
Cadlina franklinae ZMMU Op−847
Cadlina franklinae ZMMU Op−846
Cadlina franklinae ZMMU Op−857
Cadlina franklinae ZMMU Op−844
Cadlina bellburnellae ZMMU Op−872
Cadlina bellburnellae ZMMU Op−871
Cadlina bellburnellae ZMMU Op−870
